# Elucidation of Charge Contribution in Iridium-Chelated Hydrogen-Bonding Systems

**DOI:** 10.3389/fchem.2021.712698

**Published:** 2021-08-24

**Authors:** Barbora Balónová, Barry A. Blight

**Affiliations:** Department of Chemistry, University of New Brunswick, Fredericton, NB, Canada

**Keywords:** H-bonding array, charge-assisted, ligand non-innocence, non-covalent interactions, self-assembly

## Abstract

We present two iridium complexes **1H** ^**+**^ and **2H** ^**+**^ that contain cationic ligands to extend the knowledge of charge-assisted hydrogen bonding (CAHB), which counts among the strongest non-covalent bonding interactions. Upon protonation, both complexes were converted into new hydrogen-bonding arrays with various selectivity for respective H-bonding partners. This study compares the association strengths of four hydrogen-bonding co-systems, emphasizing the roles of CAHB in supramolecular systems. We determined that the cationic charge in these systems contributed up to 2.7 kJ mol^−1^ in the H-bonding complexation processes.

## Introduction

Hydrogen-bonding is a type of interaction that plays a crucial role in most branches of science (Marechal, 2007). Not surprisingly, this interaction is often used in biochemical processes (Moran et al., 2012), materials science (Chowdhury and Gillespie, 2018), and many applicative areas of supramolecular chemistry (Kuhn et al., 2010; Persch et al., 2015). The electrostatic interaction occurs between the partial positively charged hydrogen atom X-H (donor) and a partial negatively charged hydrogen acceptor atom Y, where X and Y are electronegative atoms (such as N, O, or S). In addition to hydrogen bonds (H-bonds), supramolecular systems can be reinforced by the cooperative interactions between binding partners in the assembly (Prins et al., 2001). An important example of this statement is charge-assisted H-bonding (CAHB), which can be described as an interaction of the X-H^+^···Y^−^ type, where the X-H donor belongs to the cation, and the Y acceptor belongs to the anion. Here, the charge assisted bonds X-H^+^···Y^−^, also known as a salt bridge, combine the inherent strength and directionality of the hydrogen bond with favorable localization of the ionic charges while being easily obtained via an acid-base reaction (Braga et al., 2000). Previously reported strategies for the preparation of CAHB systems often involve strategies incorporating nitrogen-based compounds (amines, amides, amidines), which can accept a proton from a carboxylic acid, for example, leading to the formation of N-H^+^···O^−^ interactions (Papoutsakis et al., 1999; Félix et al., 2000; Schmuck and Wienand, 2003) with free energies ranging from 4.0–5.2 kJ mol^−1^ (Horovitz et al., 1990).

Leigh and coworkers presented quadruple hydrogen-bonding complexes, including protonated salts with four N-H···N interactions that include an ion-dipole N-H^+^···N array (Leigh et al., 2013). These interactions can be switched on/off by the controlled addition of acid and base (Blight et al., 2011). Such configurations may be useful for designing responsive materials, such as nanofibers, gels, and supramolecular polymers. CAHBs tend to possess stronger interactions than a simple hydrogen bond due to the additional electrostatic interaction involved, resulting from one or more of the components bearing a charge (Papmeyer et al., 2016; Pop et al., 2016). Experimental deconvolution of sole-charge contribution in CAHB systems has yet to be quantified in assemblies where multiple hydrogen bonding arrays are employed. Several reports have shown that CAHB systems have found application in crystal engineering (Liu et al., 2019), synthesis of pharmaceutical salts/co-crystals (Wang et al., 2014), and in organometallic systems (Braga et al., 2004), making the elucidation of this energetic contribution critical in predicting materials properties.

**FIGURE 1:**
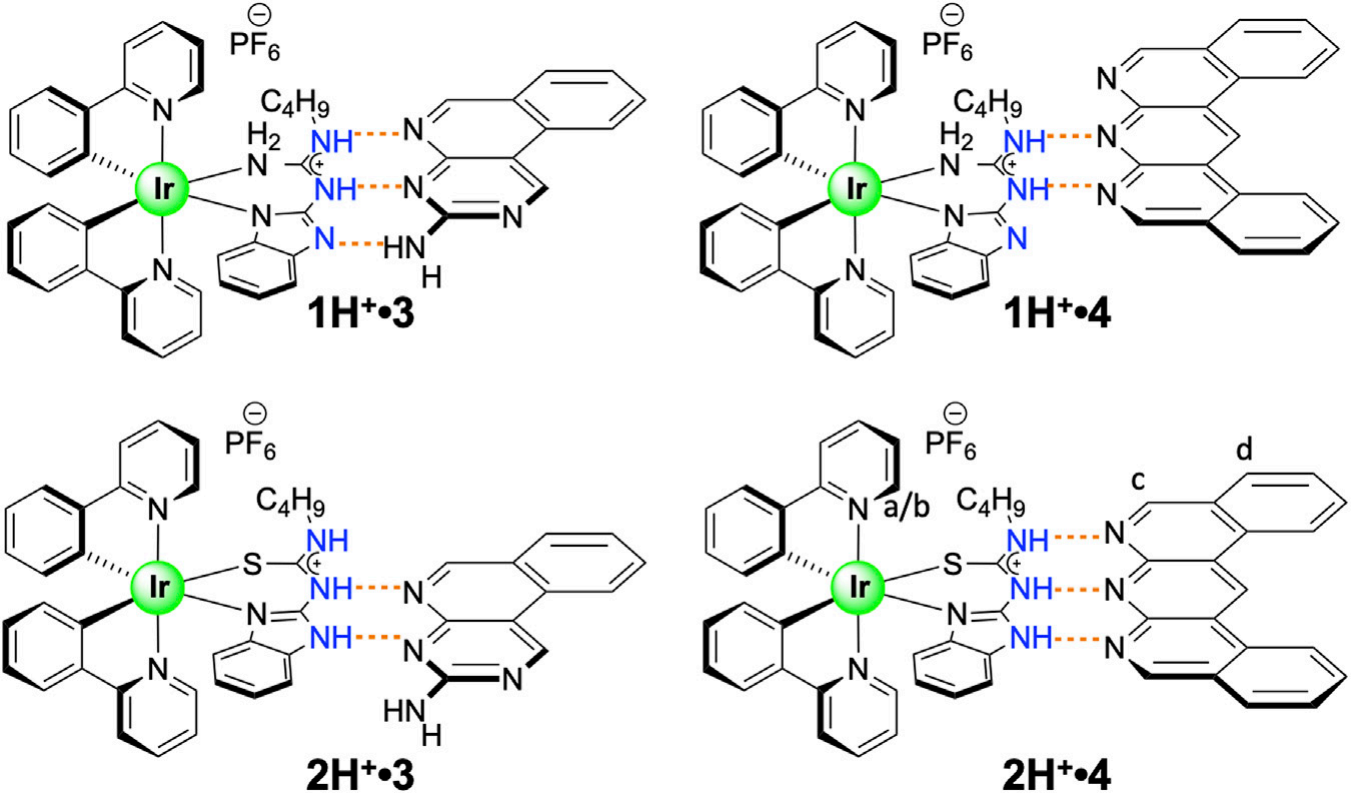
Four complementary charge-assisted H-bonding systems featured in this study.

In this study, we explore the effect of CAHB through the protonation of guanidine and thiourea-based ligands. According to the study conducted by Taylor and Kennard, N-H donors with a formal positive charge tend to form shorter bonds than uncharged N-H groups (Taylor and Kennard, 1984), which indicates a stronger association strength. Guanidinium derivatives represent a versatile functional group with unique properties (Blondeau et al., 2007; Han et al., 2008; Gale et al., 2013), and together with thiourea derivatives (Lee et al., 2002), have been widely investigated as part of the supramolecular systems. As such, we present here a comprehensive study of non-covalent self-assembly of the ionic iridium (III) complexes **1H** ^**+**^ and **2H** ^**+**^ (illustrated in Figure 1) with two different guest molecules 3 (Balónová et al., 2018) and 4 (Blight et al., 2009). These cationic complexes were found to exhibit stronger association constants than with the neutral species **1** (Balónová et al., 2018) and **2** (Balónová et al., 2020) when combined with complementary binding partners **3** and **4**. Chelation of the iridium (III) center by the guanidine and thiourea ligands eliminates any destructive rotational energy allowing us to accurately determine the contribution of the cationic charge to the association strength *via* experimentation.

## Results and Discussion

We have previously reported the synthesis and characterization data of thiourea and guanidine-based ligands used for the synthesis of iridium complexes **1H** ^**+**^ and **2H** ^**+**^ (Balónová et al., 2018; Balónová et al., 2020). Synthetic details for cationic complexes **1H** ^**+**^ and **2H** ^**+**^ are presented in the supplementary material for this article (Supplementary Section S1). Iridium *μ*-chloro-bridged dimer [Ir (ppy)_2_Cl]_2_ (ppyH = phenylpyridine) dimer was prepared by the procedure reported by Nonoyama (Nonoyama, 1974). Complex **1H** ^**+**^ was synthesized using 1-(1H-benzo [d]imidazole-2-yl)-3-butylguanidine as a ligand followed by the ligand exchange using potassium hexafluorophosphate (KPF_6_^-^) as the source of PF_6_^-^ counterion. Iridium complex **2H** ^**+**^ was synthesized by refluxing 1-(1H-benzo [d]imidazole-2-yl)-3-butylthiourea ligand with the iridium *μ*-chloro-bridged dimer [Ir (ppy)_2_Cl]_2_ in toluene, and similarly followed by the ion exchange with KPF_6_^-^ counterion for the cationic complex **2H** ^**+**^. Complexes **1H** ^**+**^ and **2H** ^**+**^ were paired with binding partners **3** and **4** (Figure 1), and association constants were determined. UV-vis absorption spectroscopy titration methods were used to measure the association constants for complexes **1H** ^**+**^ **•3/4** and **2H** ^**+**^ **•3/4**, and all data were analyzed with the program BindFit (Thordarson, 2011; Supplementary Section S5) or sivvu.org as noted. The titrations were carried out in HPLC grade CHCl_3_ with 1% of DMSO to support the solubility of binding partners **3** and **4**. The self-association (*K*_*dim*_) of compounds **3** and **4** was determined to be *K*_*dim*_ < 50 M^−1^ and considered negligible for this study.

**FIGURE 2:**
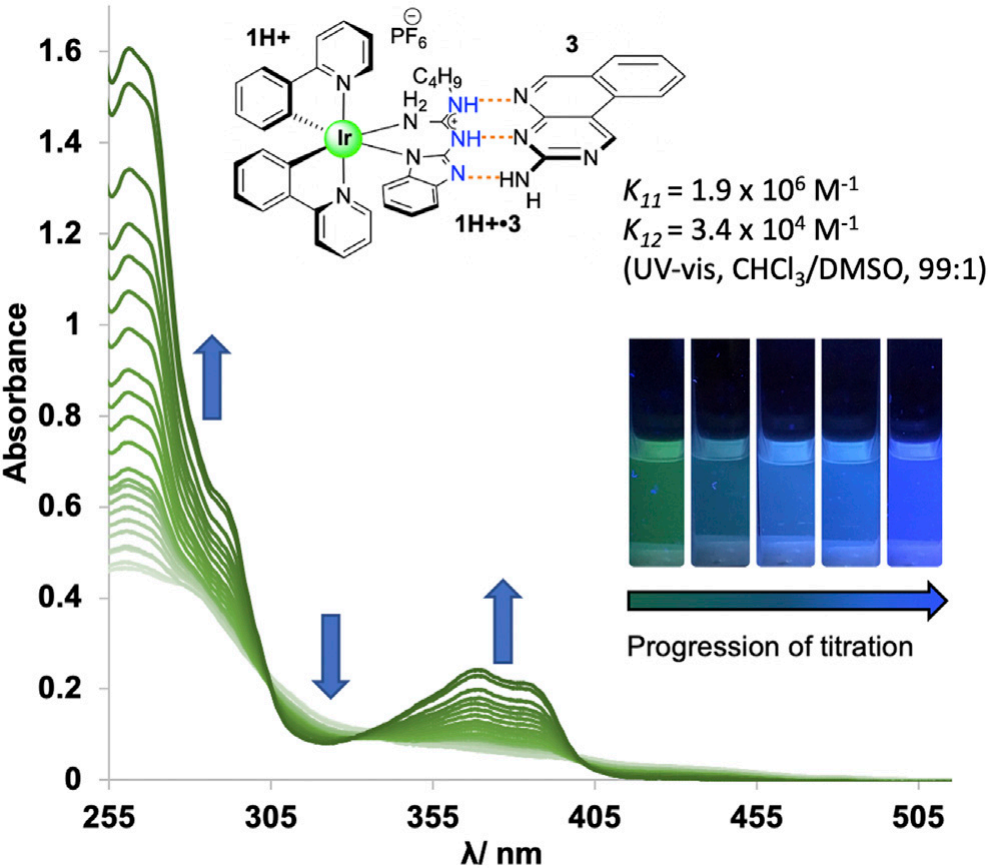
UV-vis absorbance spectra from a titration experiment (298 K) for co-system **1H** ^**+**^ **•3** in CHCl_3_/DMSO (99:1 v/v).

Cationic guanidine-based complex **1H** ^**+**^ was paired with binding partners **3** and **4**, and association constants were determined through UV-vis absorption titration studies, with results summarized in Table 1. Due to the increased acidity of NH protons in the guanidinium moiety in complex **1H** ^**+**^, higher association constants were expected for systems **1H** ^**+**^ **•3/4** in comparison to the association constants for thiourea based systems **2H** ^**+**^ **•3/4**. Gibbs free energies for all co-systems, together with the predicted energy values from the empirical model, are also presented in Table 1. Titration study for co-system **1H** ^**+**^ **•3** (Figure 2; Supplementary Section S5) revealed an increased association constants *K*_*11*_ = 1.9 × 10^6^ M^−1^ and *K*_*12*_ = 3.4 × 10^4^ M^−1^ (UV-vis, CHCl_3_/DMSO, (99:1 v/v)) in comparison to neutral system **1•3** (Table 1). To our surprise, experimental results obtained from UV-vis absorption titration studies with binding partner **4** did not align with our hypothesis. Admittedly, the association strength for protonated co-system **1H** ^**+**^ **•4** (DDD^+^-AAA array) – where protonation of the benzimidazole would lead to a DDD^+^ system, a perfect complement to **4**—did not increase compared to neutral co-system **1•4** (DDA-AAA array). As reported by Wisner and coworkers, the association strength can be decreased or increased by changing the structure of the interacting site to the other isomeric form (Linares Mendez et al., 2019). We posit that prototropy of the guanidinium ligand, made possible by the multiple basic sites that guanidine offers, gives rise to a protonated state that does not give rise to the desired DDD^+^ arrangement, but an ADD^+^ array (Supplementary Section S1), as evidenced by the lower-than-expected association constant for **1H** ^**+**^ **•4**, with a modest increase of binding strength observed for **1H** ^**+**^ **•3**.

**TABLE 1:** Experimentally determined association constants for **1H** ^**+**^ and **2H** ^**+**^ with two different guest molecules, **3** and **4**, and their neutral parent complexes.

Co-system	Association	−ΔG	Sartorius
	Constant^a^ (*K*_a_)	(kJ mol^−1^)	(kJ mol^−1^)
**1•3** ^b^	*K*_*11*_ = 9.1 × 10^5^ M^−1^	34.0	23.7
	*K*_*12*_ = 3.2 × 10^4^ M^−1^	25.7	—
**1•4** ^c^	*K*_*11*_ = 9.9 × 10^4^ M^−1^	28.5	21.6
	*K*_*12*_ = 4.2 × 10^3^ M^−1^	20.7	—
**1H** ^**+**^ **•3** ^b^	*K*_*11*_ = 1.9 × 10^6^ M^−1^	35.9	23.7
	*K*_*12*_ = 3.4 × 10^4^ M^−1^	25.9	—
**1H** ^**+**^ **•4** ^c^	*K*_*a*_ = 1.5 × 10^3^ M^−1^	18.1	21.6
**2•3** ^d^	*K*_*a*_ = 2.1 × 10^3^ M^−1^	19.0	23.7
**2•4** ^d^	*K*_*a*_ = 1.6 × 10^3^ M^−1^	18.3	21.6
**2H** ^**+**^ **•3** ^c^	*K*_*a*_ = 4.8 × 10^3^ M^−1^	21.0	21.6
**2H** ^**+**^ **•4** ^c^	*K*_*11*_ = 2.0 × 10^4^ M^−1^	24.5	35.3
	*K*_*12*_ = 8.6 × 10^3^ M^−1^	22.4	—

a Measured by UV-vis absorption spectroscopy in CHCl_3_/DMSO (99:1 v/v), 298 K.

b Data modelled using sivvu.org.

c Data modelled using Bindfit from supramolecular.org.

d Data from previously reported work (Balónová et al., 2020).

**FIGURE 3:**
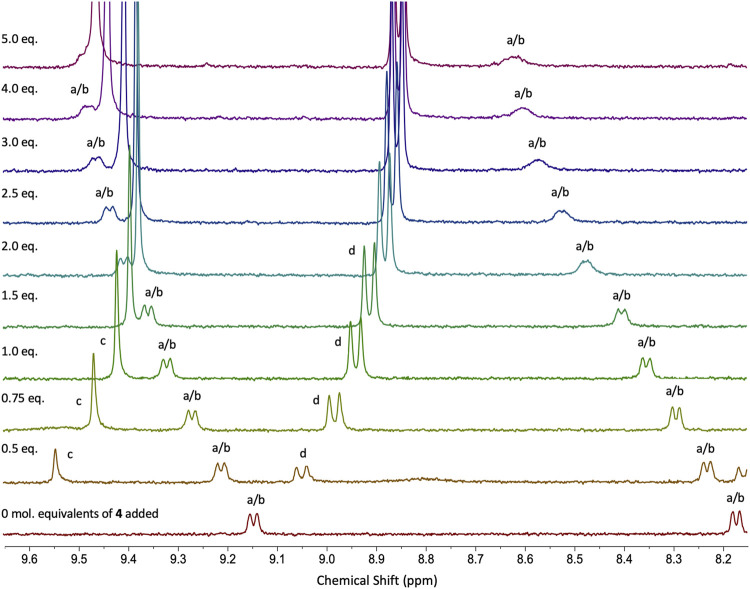
Stacked ^1^H NMR (400 MHz, 298 K) spectra from titration experiment for co-system **2H** ^**+**^ **•4** in CDCl_3_/DMSO-*d*_6_ (99:1 v/v). **4** (c = 1 × 10^−3^ M) was titrated into a solution of **2H** ^**+**^ (c = 1 × 10^−4^ M) in CDCl_3_/DMSO-*d*_6_ (99:1 v/v).cs.

Compound **2H** ^**+**^ was separately paired with guests **3** and **4**, and their interactions were examined through UV-vis spectroscopic analysis (Supplementary Section S5) to quantify their respective association constants and compared with neutral systems **2•3** and **2•4** (Table 1). The co-system **2H** ^**+**^ **•3** can be described as a double bonding DD^+^-AA motif with three attractive and one repulsive secondary interaction within the structure. The strength of this association was assessed via UV-vis absorption titration of **2H** ^**+**^ with **3** in CHCl_3_/DMSO (99:1 v/v), revealing an association constant *K*_*a*_ = 4.8 × 10^3^ M^−1^ ± 0.4% and the binding energy of -21.6 kJ mol^−1^, which is almost identical to the value obtained from the Sartorius empirical model that assigns weighted interaction values as the number of interactions increase (Sartorius and Schneider, 1996). This value is almost doubled compared to the neutral co-system **2•3**, which can be explained by the increased number of attractive secondary interactions and electrostatic-charge assistance contributing to the stability and binding energy of the **2H** ^**+**^ **•3** system (Table 1). The association constant for co-system **2H** ^**+**^ **•4** was also investigated, and according to the strong influence of secondary interactions, the complementary DDD^+^-AAA system was predicted to be among the most stable arrays presented in this study. As has been previously investigated (and noted above), the binding strength is maximized if all the donor atoms are located on one component and all acceptor atoms are on the binding partner (Jorgensen and Pranata, 1990; Pranata et al., 1991). The planar compound **4** has been previously reported to improve stability and give rise to high association constants in triple DDD-AAA systems (Blight et al., 2009). The neutral complex **2** formed a double H-bonding DD-AA array with **4** (*K*_*a*_ = 1.6 × 10^3^ M^−1^ ± 0.1%; Figure 2), and through simple protonation, the multiplicity was increased to triple H-bonding DDD^+^-AAA system **2H** ^**+**^ **•4**. Multiple examples of DDD-AAA complexes have been reported to date (only two DDD^+^), but none of them considered thiourea ligands as binding partners in the assemblies (Balónová et al., 2020; Djurdjevic et al., 2007). Addition of **4** to **2H** ^**+**^ in CHCl_3_/DMSO (99:1 v/v) was monitored by UV-vis absorption titration analysis and association constants *K*_*11*_ = 2.0 × 10^4^ M^−1^ ± 0.1%, *K*_*12*_ = 8.6 × 10^3^ M^−1^ ± 0.2% for co-system **2H** ^**+**^ **•4** were determined (confirmed by ^1^H NMR; Figure 3; Supplementary Sections S4, S5). Compared to the neutral co-system **2•4**, protonation of complex **2** resulted in ∼ 12-fold increase in the association constant when combined with compound **4** in CHCl_3_/DMSO (99:1 v/v). ^1^H NMR titrations illustrate the putative interactions between **2H** ^**+**^ and **4** with protons *a/b* of **2H** ^**+**^ (*o*-protons of both pyridine moieties) being shifted down-field by approx. 0.5 ppm and protons *c* and *d* of **4** showing a reciprocal shift up-field as its concentration is increased in the presence of host **2H** ^**+**^.

**FIGURE 4:**
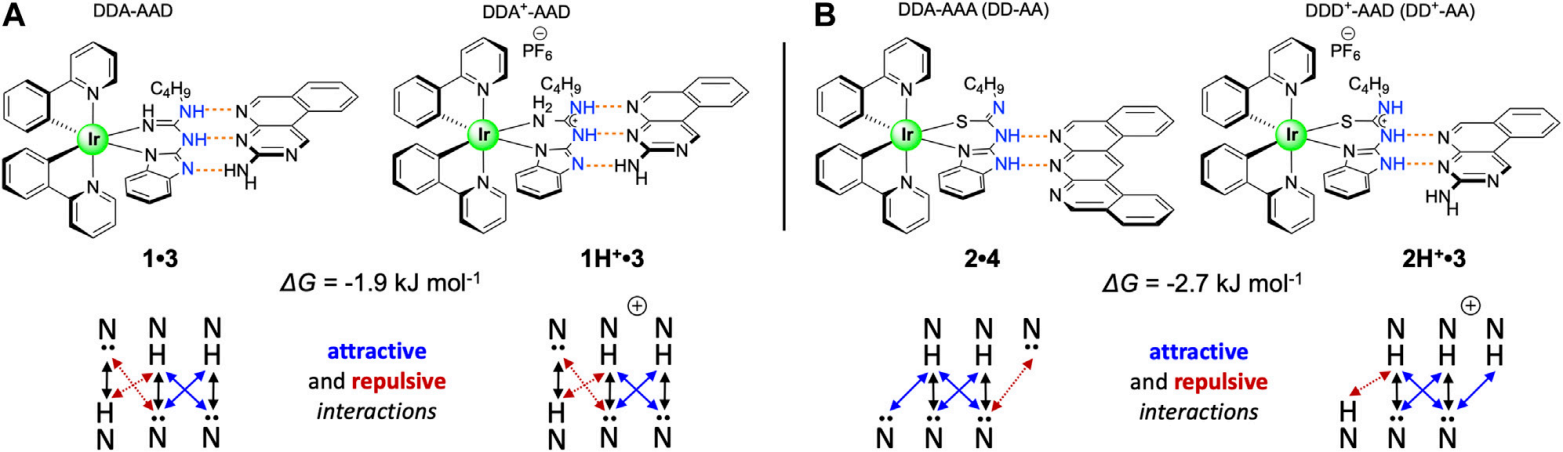
Structural comparison of co-systems **(A) 1•3** and **1H** ^**+**^ **•3** and **(B) 2•4** and **2H** ^**+**^ **•3** toward experimentally elucidating the charge contribution to these association events.

Based on these results, we were able to use an empirical approach to calculate the contribution of charge to the association strength for two of our systems. Protonation of **1** gives rise to the **1H** ^**+**^, where complex prototropy (Supplementary Section S1) inhibits identification of the extra proton location. Given that there is a large increase in *K*_a_ for **1H** ^**+**^ **•3** and not for **1H** ^**+**^ **•4,** which would represent a DDD^+^-AAA array, we propose that **1H** ^**+**^ **•3** exists as an ADD^+^ array (vs DDD^+^) complemented by **3** (DAA; Figure 4A), which allows us to directly compare its *K*_a_ with that of **1•3** (ADD-DAA) given that they have the same number of primary H-bonds and secondary electrostatic interactions. Comparing the neutral guanidine-based co-system **1•3** (ΔG_1:1_ = −34.0 kJ mol^−1^) with the cationic **1H** ^**+**^ **•3** (ΔG_1:1_–35.9 kJ mol^−1^), we calculated the overall charge contribution to the association, as the difference in Gibbs free energy, to be −1.9 kJ mol^−1^ (−0.45 kcal mol^−1^). As presented in Figure 4B, thiourea-based systems **2•4** and **2H** ^**+**^ **•3** empirically have the same number of primary hydrogen bonds and attractive/repulsive secondary interactions within the structures, assuming that the different secondary electrostatic interactions contribute equally. Based on this structural arrangement, the charge contribution was calculated. From the comparison of neutral thiourea-based co-system **2•4** (ΔG = −18.3 kJ mol^−1^) with the cationic **2H** ^**+**^ **•3** (ΔG = −21.0 kJ mol^−1^) we calculated the charge contribution in this instance to be −2.7 kJ mol^−1^ (−0.65 kcal mol^−1^). We note the difference in values in the two different systems and acknowledge that these are using the *K*_1:1_ values from these equilibria to do this comparison. We posit that the competitive 1:2 equilibria will interfere with determining a wholly discrete cation contribution. However, to the best of our knowledge, the discrete charge contribution to association strength in H-bonding arrays has never been determined before. If comparing the Gibbs free energy of charge contribution to salt bridges as determined by Horowitz and coworkers (4.0–5.2 kJ mol^−1^; Horovitz et al., 1990) the charge contributions elucidated in this study are in agreement, given that the present study includes only one of the charged partners.

In summary, we prepared two new cationic iridium (III) complexes **1H** ^**+**^ and **2H** ^**+**^. Both complexes **1H** ^**+**^ and **2H** ^**+**^ represent rare examples of charged complexes where the ancillary ligand carries the formal charge (ligand non-innocence). This work further examined the self-assembly of complexes **1H** ^**+**^ and **2H** ^**+**^ with guest molecules **3** and **4**, respectively, to determine the charge contribution to the association strength. Guanidine based complex **1H** ^**+**^ with component **3** in DDA-AAD alignment represents the strongest H-bonding system (*K*_*11*_ = 1.9 × 10^6^ M^−1^ and *K*_*12*_ = 3.4 × 10^4^ M^−1^, UV-vis, CHCl_3_/DMSO, (99:1 v/v)) in this study due to increased acidity of NH protons in the cationic **1H** ^**+**^ system, a 2-fold increase over its neutral system. In addition, simple protonation of thiourea-based complex **2** results in a ∼12-fold increase in the association strength of co-system **2H** ^**+**^ **•4** in comparison to its neutral version **2•4**. Furthermore, from UV-vis absorption titration studies, we were able to determine the contribution of the charge to the association strength by comparing neutral systems **1•3** and **2•4** with their respective complements **1H** ^**+**^ **•3** and **2H** ^**+**^ **•3** to be −1.9 kJ mol^−1^ (−0.45 kcal mol^−1^) and −2.7 kJ mol^−1^ (−0.65 kcal mol^−1^), respectively). Elucidating the energetics of CAHB interactions will contribute to developing empirical models that allow for more accurate prediction of system dynamics. Based on these results, incorporating CAHB interactions into H-bonding arrays can increase association strengths, leading to higher-order materials and a significant role in more competitive and complex systems.

## Data Availability

The datasets presented in this study can be found in online repositories. The names of the repository/repositories and accession number(s) can be found below: https://doi.org/10.25545/6TZWCG.

## References

[B1] Balónová B. Martir D. R. Clark E. R. Shepherd H. J. Zysman-Colman E. Blight B. A. (2018). Influencing the Optoelectronic Properties of a Heteroleptic Iridium Complex by Second-Sphere H-Bonding Interactions. Inorg. Chem. 57, 8581–8587. 10.1021/acs.inorgchem.8b01326 29969251

[B2] Balónová B. Shepherd H. J. Serpell C. J. Blight B. A. (2020). IrIII as a Strategy for Preorganisation in H-Bonded Motifs. Supramolecular Chem. 32, 1–12. 10.1080/10610278.2019.1649674

[B3] Blight B. A. Camara-Campos A. Djurdjevic S. Kaller M. Leigh D. A. McMillan F. M. (2009). AAA−DDD Triple Hydrogen Bond Complexes. J. Am. Chem. Soc. 131, 14116–14122. 10.1021/ja906061v 19746984

[B4] Blight B. A. Hunter C. A. Leigh D. A. McNab H. Thomson P. I. T. (2011). An AAAA-DDDD Quadruple Hydrogen-Bond Array. Nat. Chem 3, 244–248. 10.1038/nchem.987 21336332

[B5] Blondeau P. Segura M. Pérez-Fernández R. de Mendoza J. (2007). Molecular Recognition of Oxoanions Based on Guanidinium Receptors. Chem. Soc. Rev. 36, 198–210. 10.1039/B603089K 17264923

[B6] Braga D. Maini L. Grepioni F. De Cian A. Fe´lix O. Fischer J. (2000). Charge-assisted N-H(+)···O(-) and O-H···O(-) Hydrogen Bonds Control the Supramolecular Aggregation of Ferrocenedicarboxylic Acid and Bis-Amidines. New J. Chem. 24, 547–553. 10.1039/B002061N

[B7] Braga D. Polito M. Grepioni F. (2004). Novel Organometallic Building Blocks for Molecular Crystal Engineering. 3. Synthesis, Characterization, and Hydrogen Bonding of the Crystalline Mono- and Bis-Amide Derivatives of [CoIII(η5-C5h4-COOH)2]+and of the Cationic Zwitterion [CoIII(η5-C5H4CONHC5H4NH)(η5-C5H4COO)]+. Cryst. Growth Des. 4, 769–774. 10.1021/cg049942w

[B8] Chowdhury S. C. Gillespie J. W. (2018). A Molecular Dynamics Study of the Effects of Hydrogen Bonds on Mechanical Properties of Kevlar crystal. Comput. Mater. Sci. 148, 286–300. 10.1016/j.commatsci.2018.02.055

[B9] Djurdjevic S. Leigh D. A. McNab H. Parsons S. Teobaldi G. Zerbetto F. (2007). Extremely Strong and Readily Accessible AAA−DDD Triple Hydrogen Bond Complexes. J. Am. Chem. Soc. 129, 476–477. 10.1021/ja067410t 17226995

[B10] Félix O. Hosseini M. W. De Cian A. Fischer J. (2000). Crystal Engineering of 2-D Hydrogen Bonded Molecular Networks Based on the Self-Assembly of Anionic and Cationic Modules. Chem. Commun. 4, 281–282. 10.1039/A909093B

[B11] Gale P. A. Pérez-Tomás R. Quesada R. (2013). Anion Transporters and Biological Systems. Acc. Chem. Res. 46, 2801–2813. 10.1021/ar400019p 23551251

[B12] Han J. Yau C.-W. Lam C.-K. Mak T. C. W. (2008). Designed Supramolecular Assembly of Hydrogen-Bonded Anionic Rosette Layers. J. Am. Chem. Soc. 130, 10315–10326. 10.1021/ja802425q 18611020

[B13] Horovitz A. Serrano L. Avron B. Bycroft M. Fersht A. R. (1990). Strength and Co-operativity of Contributions of Surface Salt Bridges to Protein Stability. J. Mol. Biol. 216, 1031–1044. 10.1016/S0022-2836(99)80018-7 2266554

[B14] Jorgensen W. L. Pranata J. (1990). Importance of Secondary Interactions in Triply Hydrogen Bonded Complexes: Guanine-Cytosine vs Uracil-2,6-Diaminopyridine. J. Am. Chem. Soc. 112, 2008–2010. 10.1021/ja00161a061

[B15] Kuhn B. Mohr P. Stahl M. (2010). Intramolecular Hydrogen Bonding in Medicinal Chemistry. J. Med. Chem. 53, 2601–2611. 10.1021/jm100087s 20175530

[B16] Lee D. H. Lee H. Y. Hong J.-I. (2002). Anion Sensor Based on the Indoaniline-Thiourea System. Tetrahedron Lett. 43, 7273–7276. 10.1016/S0040-4039(02)01455-7

[B17] Leigh D. A. Robertson C. C. Slawin A. M. Z. Thomson P. I. T. (2013). AAAA-DDDD Quadruple Hydrogen-Bond Arrays Featuring NH···N and CH···N Hydrogen Bonds. J. Am. Chem. Soc. 135, 9939–9943. 10.1021/ja404504m 23763627

[B18] Linares Mendez I. J. Pleizier J. S. Wang H.-B. Wisner J. A. (2018). 1 H NMR-Based Method for the Determination of Complexation Equilibrium Parameters and Chemical Shifts in a Hydrogen-Bonded System with Dynamic Composition. J. Phys. Org. Chem. 31, e3805. 10.1002/poc.3805

[B19] Liu L. Zou D. Zhang Y. Zhang D. Zhang Y. Zhang Q. (2019). Assembly of Three Pharmaceutical Salts/Cocrystals of Tetrahydroberberine with Sulfophenyl Acids: Improving the Properties by Formation of Charge-Assisted Hydrogen Bonds. New J. Chem. 43, 4886–4894. 10.1039/C9NJ00131J

[B20] Marechal Y. (2007). The Hydrogen Bond and the Water Molecule: The Physics and Chemistry of Water, Aqueous and Bio media. Amsterdam, Netherlands: Elsevier Science & Technology.

[B21] Moran L. A. Horton H. R. Scrimgeour K. G. Perry M. D. (2012). Principles of Biochemistry. 5th ed. New York: Pearson Education.

[B22] Nonoyama M. (1974). Benzo[h]quinolin-10-yl-NIridium(III) Complexes. Bcsj 47, 767–768. 10.1246/bcsj.47.767

[B23] Papmeyer M. Vuilleumier C. A. Pavan G. M. Zhurov K. O. Severin K. (2016). Molecularly Defined Nanostructures Based on a Novel AAA-DDD Triple Hydrogen-Bonding Motif. Angew. Chem. 128, 1717–1721. 10.1002/anie.20151042310.1002/ange.201510423 26695538

[B24] Papoutsakis D. Kirby J. P. Jackson J. E. Nocera D. G. (1999). From Molecules to the Crystalline Solid: Secondary Hydrogen-Bonding Interactions of Salt Bridges and Their Role in Magnetic Exchange. Chem. Eur. J. 5, 1474–1480. 10.1002/(SICI)1521-3765(19990503)5:5<1474::AID-CHEM1474>3.0.CO;2-T

[B25] Persch E. Dumele O. Diederich F. (2015). Molecular Recognition in Chemical and Biological Systems. Angew. Chem. Int. Ed. 54, 3290–3327. 10.1002/anie.201408487 25630692

[B26] Pop L. Hadade N. D. van der Lee A. Barboiu M. Grosu I. Legrand Y.-M. (2016). Occurence of Charge-Assisted Hydrogen Bonding in Bis-Amidine Complexes Generating Macrocycles. Cryst. Growth Des. 16, 3271–3278. 10.1021/acs.cgd.6b00246

[B27] Pranata J. Wierschke S. G. Jorgensen W. L. (1991). OPLS Potential Functions for Nucleotide Bases. Relative Association Constants of Hydrogen-Bonded Base Pairs in Chloroform. J. Am. Chem. Soc. 113, 2810–2819. 10.1021/ja00008a002

[B28] Prins L. J. Reinhoudt D. N. Timmerman P. (2001). Non-covalent Synthesis Using Hydrogen Bonding. Angew. Chem. Int. Ed. 40, 2382–2426. 10.1002/1521-3773(20010702)40:13<2382::AID-ANIE2382>3.0.CO;2-G 11443654

[B29] Sartorius J. Schneider H.-J. (1996). A General Scheme Based on Empirical Increments for the Prediction of Hydrogen-Bond Associations of Nucleobases and of Synthetic Host-Guest Complexes. Chem. Eur. J. 2, 1446–1452. 10.1002/chem.19960021118

[B30] Schmuck C. Wienand W. (2003). Highly Stable Self-Assembly in Water: Ion Pair Driven Dimerization of a Guanidiniocarbonyl Pyrrole Carboxylate Zwitterion. J. Am. Chem. Soc. 125, 452–459. 10.1021/ja028485+ 12517158

[B31] Taylor R. Kennard O. (1984). Hydrogen-Bond Geometry in Organic Crystals. Acc. Chem. Res. 17, 320–326. 10.1021/ar00105a004

[B32] Thordarson P. (2011). Determining Association Constants from Titration Experiments in Supramolecular Chemistry. Chem. Soc. Rev. 40, 1305–1323. 10.1039/c0cs00062k 21125111

[B33] Wang H. Gurau G. Shamshina J. Cojocaru O. A. Janikowski J. MacFarlane D. R. (2014). Simultaneous Membrane Transport of Two Active Pharmaceutical Ingredients by Charge Assisted Hydrogen Bond Complex Formation. Chem. Sci. 5, 3449–3456. 10.1039/C4SC01036A

