# Supplementary material for: Elucidation of Charge Contribution in Iridium-Chelated Hydrogen-Bonding Systems
Source: Front Chem. 2021 Aug 24;9:712698. doi: 10.3389/fchem.2021.712698 (PMC8421767; doi:10.3389/fchem.2021.712698)
Supplement: Supplementary file 1 [file Datasheet1.pdf]

Elucidation of Charge Contribution in Iridium-Chelated Hydrogen-Bonding Systems

Barbora Balónová, Barry A. Blight*

[a]Department of Chemistry, University of New Brunswick, Fredericton, Canada E3B 5A3

Supplementary Material

Table of Contents

**S1: Synthetic Procedures and Characterization Data**	S2-S3
**S2: Copies of ^{1}H, ^{13}C NMR and Mass Spectra**	S4-S6
**S3: Elemental Analysis**	S7-S8
**S4: NMR Data**	S9-S11
**S5: UV-vis Absorption Data**	S12-S17
**References**	S18

S1: Synthetic Procedures and Characterization Data

Synthesis of complex 1H+

Iridium dimer complex $[Ir(ppy)_2Cl]_2$[S1] (68 mg, 6.4 x 10^{-5} mol) and 1-(1H benzo[d]imidazol-2-yl)-3-butylguanidine[S2] (2.5 equiv.) were added to 15 mL of dry toluene. The reaction mixture was refluxed for 24 h under N_2 atmosphere. The reaction was cooled and a saturated solution of NH_4PF_6 in MeOH was added in excess while stirring (~2 mL). The reaction mixture was stirred overnight at room temperature and the solvent was removed under reduced pressure. The sample was dissolved in DCM and extracted with water (2 x 20 mL) to remove the excess of NH_4PF_6. Further purification included column chromatography (silica gel, DCM: MeOH, 20:1). The title compound **1H+** was obtained as yellow powder in 59% yield.

^{1}H NMR (400 MHz, 298 K, $CDCl_3$): δ 9.38 (s, 1H), 8.61 (d, *J* = 5.3 Hz, 1H), 8.23 (s, 1H), 8.09 (d, *J* = 5.7 Hz, 1H), 7.92 (d, *J* = 8.0 Hz, 1H), 7.84 – 7.76 (m, 2H), 7.72 (dd, *J* = 7.4, 1.1 Hz, 1H), 7.61 (t, *J* = 7.4 Hz, 2H), 7.26 (s, 1H), 7.24 (s, 1H), 7.18 – 7.13 (m, 1H), 7.08 – 7.02 (m, 2H), 6.95 (dt, *J* = 10.6, 7.3 Hz, 2H), 6.86 – 6.70 (m, 3H), 6.40 (d, *J* = 7.4 Hz, 1H), 6.19 (t, *J* = 8.0 Hz, 2H), 5.36 (t, *J* = 4.9 Hz, 1H), 4.79 (s, 1H), 3.00-2.84 (m, 2H), 1.47-1.43 (m, 2H), 1.26-1.23 (m, 2H), 0.82 (t, *J* = 7.3 Hz, 3H).

^{13}C NMR (100 MHz, 298K, $CDCl_3$): δ 169.02, 168.06, 151.55, 149.80, 149.27, 148.57, 144.48, 144.23, 143.76, 140.19, 137.33, 132.90, 131.98, 131.05, 129.99, 124.72, 124.26, 123.46, 123.01, 122.26, 122.13, 121.65, 119.15, 117.77, 110.83, 77.16, 41.17, 30.36, 19.83, 13.72.

^{31}P NMR (162 MHz, $CDCl_3$) δ -132.49, -134.32, -138.74, -143.16, -147.58, -152.00, -154.26.

EI-MS m/z calcd.: 732.24, **Found**: 732.2438.

Anal. calcd. for $C_{34}H_{33}F_6IrN_7P$: C,46.57; H,3.59; N, 11.18. **Found**: C, 46.43; H, 3.59; N, 11.31.

Synthesis of complex 2H+

Iridium dimer complex $[Ir(ppy)_2Cl]_2$[S1] (202 mg, 3.77 x 10^{-4} mol) and 1 (1Hbenzo[d]imidazol-2-yl)-3-butylthiourea[S3] (116 mg, 0.471 mmol) were added in ~ 15 mL of dry toluene. Nitrogen was bubbled through the mixture for 15 min then the reaction was heated to reflux for 24 h and then cooled to room temperature. Solvent was removed under reduced pressure and the sample was re-dissolved in dichloromethane and saturated solution of KPF_6 (~10 equiv. in MeOH) was added dropwise while stirring. Total volume added ~ 3 mL. The solution was stirred for 21 h at room temperature and then extracted with dichloromethane and water, organic phases were combined, dried over magnesium sulfate and solvent was removed under reduced pressure. The product (yellow powder) was dried on Schlenk line under vacuum for 24 h. TLC control revealed one bright spot for product (Rf = 0.53, DCM: MeOH, 10:0.5). The product was further purified through precipitation from solvent mixture of dichloromethane and hexane

and dried in vacuum for 48 h. The final product **2H+** was obtained as a yellow powder in 47% yield.

^{1}H NMR (400 MHz, 298K, $CDCl_3$) δ (ppm): 9.50 (bs, 1H), 9.14 (d, 1H), 8.18 (d, 1H), 7.96 (d, 1H), 7.83-7.71 (m, *J* = 24.9 Hz, 3H), 7.64-7.55 (m, *J* = 29.5 Hz, 2H), 7.34 (bs, 1H), 7.16 (m, 1H), 7.07 (m, 2H), 6.97-6.91 (m, 2H), 6.86 (t, 1H), 6.81-6.74 (m, 2H), 6.53 (d, 1H), 6.36 (d, 1H), 6.21 (d, 1H), 3.49 (m, 2H), 1.59 (m, 2H), 1.32 (m, 2H), 0.90 (t, 3H).

^{13}C NMR (101 MHz, 298K, $CDCl_3$) δ (ppm): 172.73, 168.77, 168.13, 153.65, 150.39, 150.07, 145.88, 143.81, 140.46, 137.70, 137.43, 131.89, 131.67, 130.97, 130.19, 130.15, 124.72, 124.38, 124.12, 123.33, 122.71, 122.65, 122.51, 122.21, 119.57, 119.36, 118.57, 111.08, 77.36, 45.99, 30.15, 19.92, 13.73.

^{31}P NMR (121 MHz, $CDCl_3$) δ (ppm): -148.30, -154.62, -160.51, -166.40, -172.57.

HRMS m/z calcd.: 749.2033. **Found**: 749.2028 [M+].

Anal. calcd. for $C_{34}H_{32}F_6IrN_6SP$: C, 46.45; H, 3.55; N, 9.29. **Found**: C, 46.03; H, 3.63; N, 9.49.

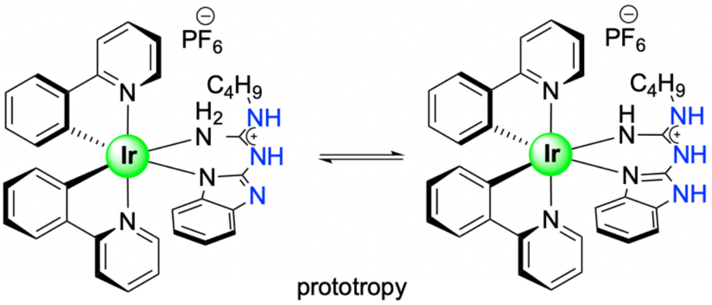

Figure S1. Illustrating prototropy of **1H**$^{+}$ where the H-bonding arrays can equilibrate between a ADD and DDD array.

S2: Copies of ^{1}H and ^{13}C NMR and Mass Spectra

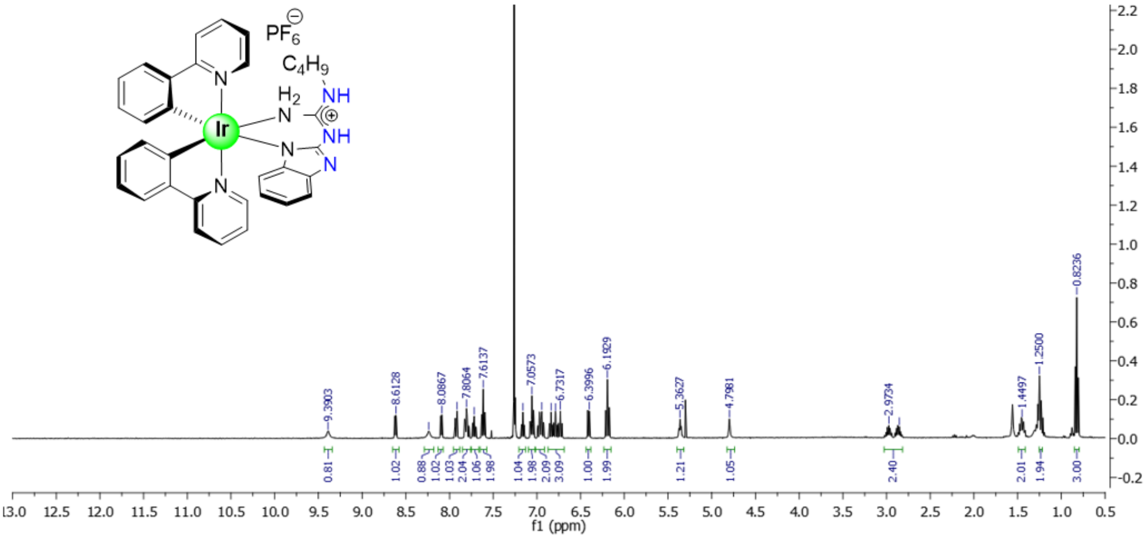

Figure S2. ^{1}H (400 MHz) NMR spectrum of **1H**$^{+}$ in chloroform-*d*, 298 K.

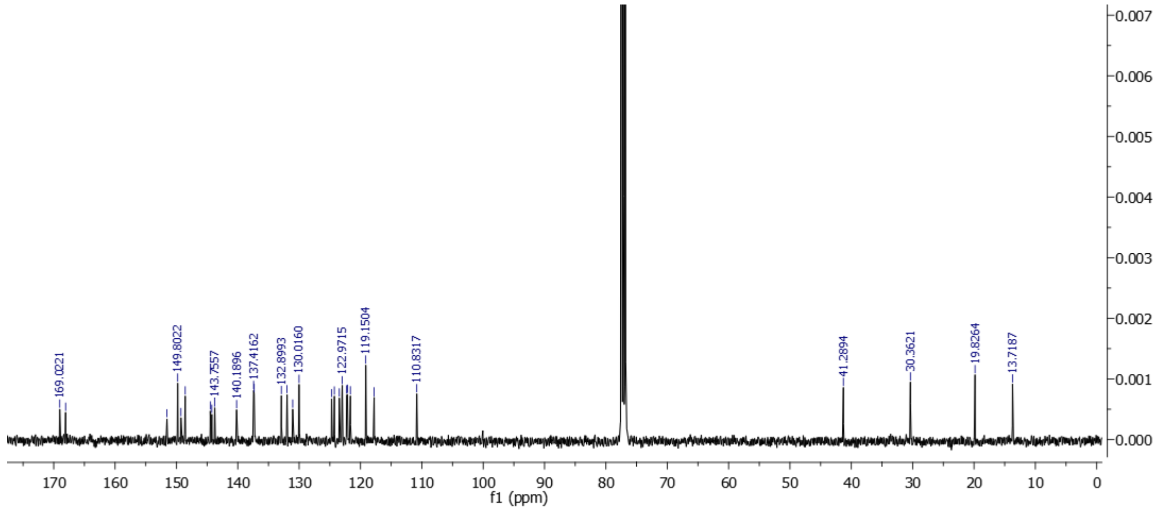

Figure S3. ^{13}C (100 MHz) NMR spectrum of **1H**$^{+}$ in chloroform-*d*, 298 K.

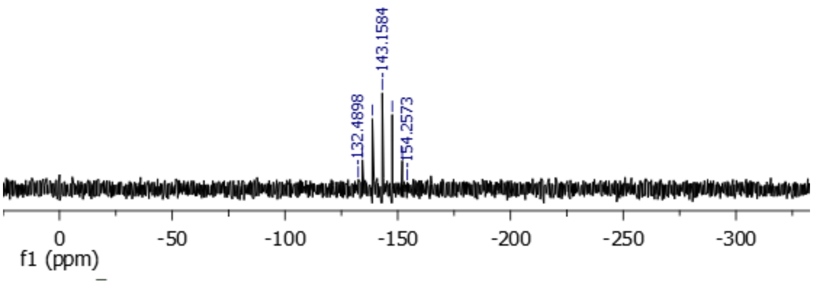

Figure S4. ^{31}P (162 MHz) NMR spectrum of **1H**$^{+}$ in chloroform-*d*, 298 K.

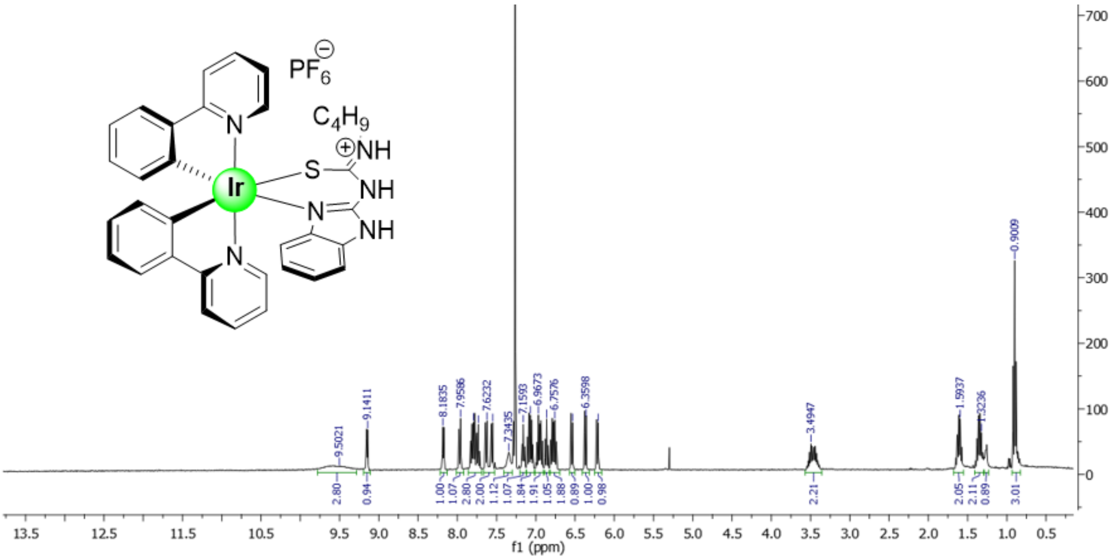

Figure S5. ^{1}H (400 MHz) NMR spectrum of **2H^{+}** in chloroform-*d*, 298 K.

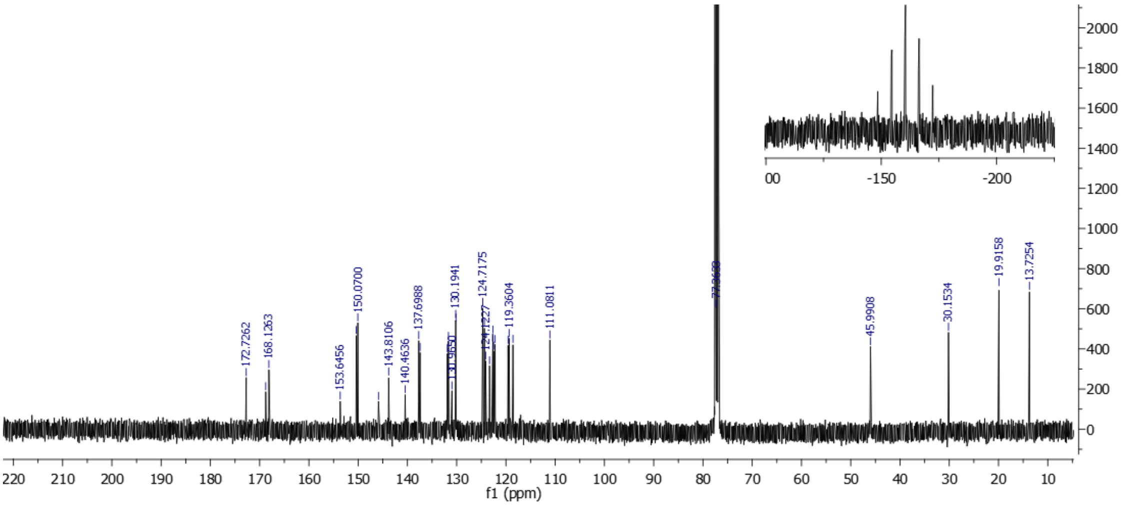

Figure S6. ^{13}C (100 MHz) NMR spectrum with inset of ^{31}P NMR spectrum (top right corner) of **2H^{+}** measured in chloroform-*d.*

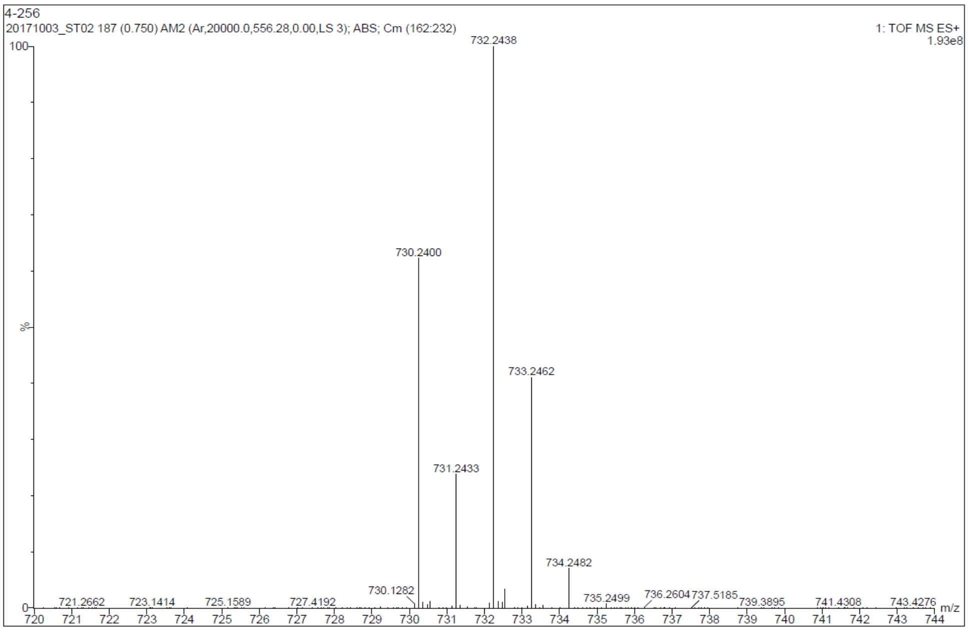

Figure S7. Mass spectrum of iridium complex **1H**$^+$.

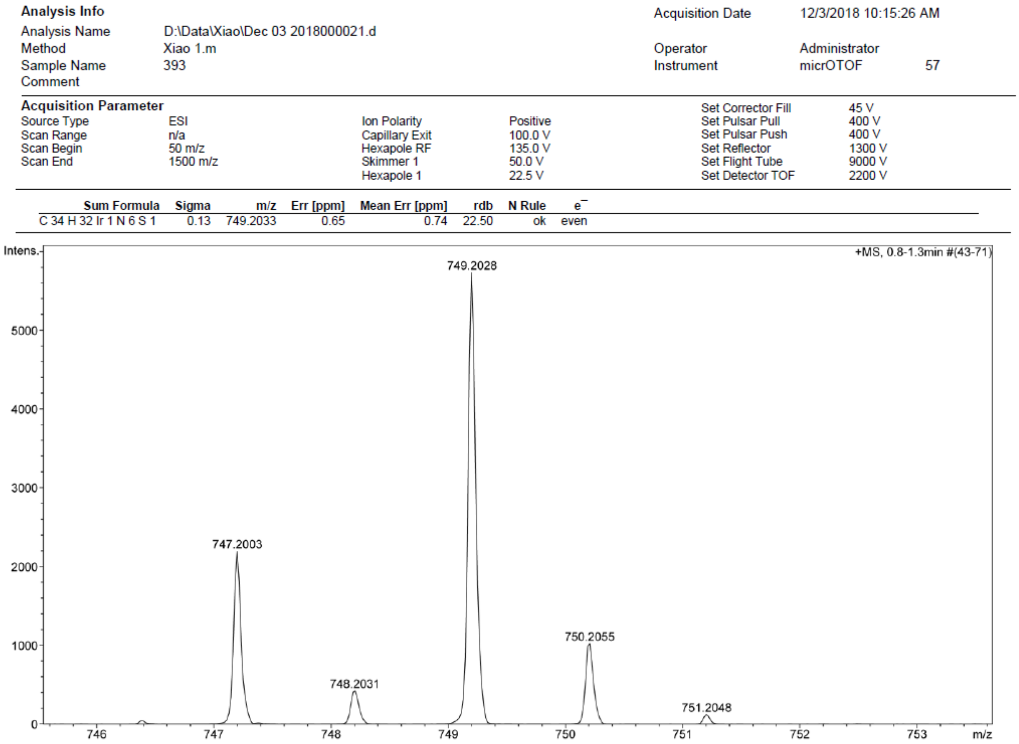

Figure S8. Mass spectrum of iridium complex **2H**$^+$.

S3: Elemental Analysis

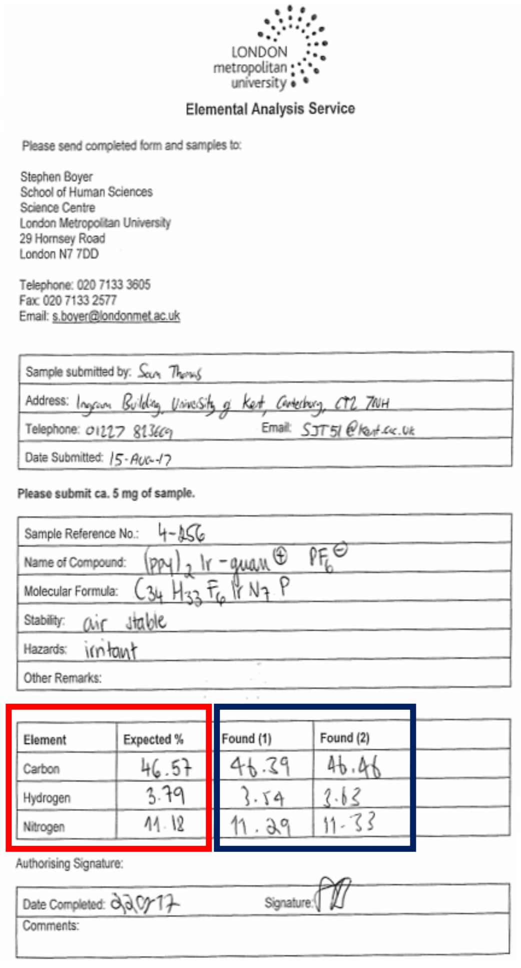

LONDON metropolitan university

Elemental Analysis Service

Please send completed form and samples to:

Stephen Boyer
School of Human Sciences
Science Centre
London Metropolitan University
29 Hornsey Road
London N7 7DD

Telephone: 020 7133 3605
Fax: 020 7133 2577
Email: s.boyer@londonmet.ac.uk

Sample submitted by: Sam Thomas
Address: Ingram Building, University of Kent, Canterbury, CT2 7NH
Telephone: 01227 813669 Email: SJT51@kent.ac.uk
Date Submitted: 15-Aug-17

Please submit ca. 5 mg of sample.

Sample Reference No.: 4-256
Name of Compound: $(ppy)_2Ir$-guan$^{\oplus}$ $PF_6^{\ominus}$
Molecular Formula: $C_{34}H_{33}F_6IrN_7P$
Stability: air stable
Hazards: irritant
Other Remarks:

Element	Expected %	Found (1)	Found (2)
Carbon	46.57	46.39	46.46
Hydrogen	3.79	3.54	3.63
Nitrogen	11.18	11.29	11.33

Authorising Signature:

Date Completed: 22/08/17 Signature: [illegible]
Comments:

Figure S9. Copy of elemental analysis for compound **1H**$^+$. Expected values highlighted in red, measured in blue.

SAINT MARY'S UNIVERSITY SINCE 1802 | FACULTY OF SCIENCE
One University. One World. Yours.

CENTRE FOR ENVIRONMENTAL ANALYSIS AND REMEDIATION
Saint Marys University, Halifax NS B3H 3C3. Science Building Room 501
Phone (902)420-5135 Email: Patricia.Granados@smu.ca

CHN Analyzer, Perkin Elmer 2400 Series II

User's Name Barbora Balonova
Analyzed by: Patricia Granados
Date: 05-Dec-18

Standard Calibration Acetanilide
Supplier Perkin Elmer

Expected Values		Carbon %	Hydrogen %	Nitrogen %
QC:Cychlohexanone-2,4-dinitro-phenylhydrazone		51.79	5.07	20.14
BB387		78.51	4.76	5.09
BB393		46.46	3.55	9.29

Quality Control Standard Results

Sample Name	Weight mg	Carbon %	Hydrogen %	Nitrogen %
QC	3.363	52.09	5.08	20.16
	3.368	51.62	4.97	20.06

Sample Results

Sample Name	Weight mg	Carbon %	Hydrogen %	Nitrogen %
BB387	3.169	78.26	4.67	5.08
BB387b	2.700	78.29	4.58	5.08
BB393	2.787	46.16	3.66	9.49
BB393b	2.553	45.90	3.59	9.49

Figure S10. Copy of elemental analysis for compound **2H**$^+$. Expected values highlighted in red, measured in blue (sample name BB393).

S4: NMR Data

^{1}H NMR titrations were performed in solutions of $CDCl_3$/ DMSO-d_6 (99:1) with small aliquots of guest (**3** or **4**) added to host (**1**, **1H$^+$**, **2**, or **2H$^+$**). Bindfit modelling was only applied to **2H$^+$•4** because of complex precipitation as the titrations concluded. UV-Vis titrations were then performed at lower concentrations to remedy this problem.

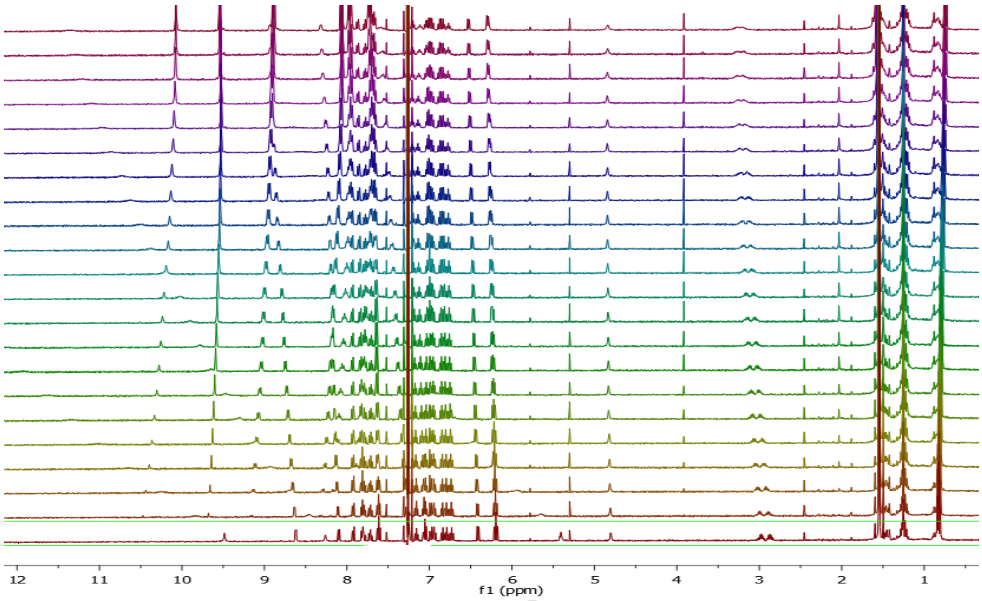

Figure S11. Stacked ^{1}H NMR (400 MHz, 298 K) from titration experiment for co-system **1H$^+$•4** in $CDCl_3$/ DMSO-d_6 (99:1).

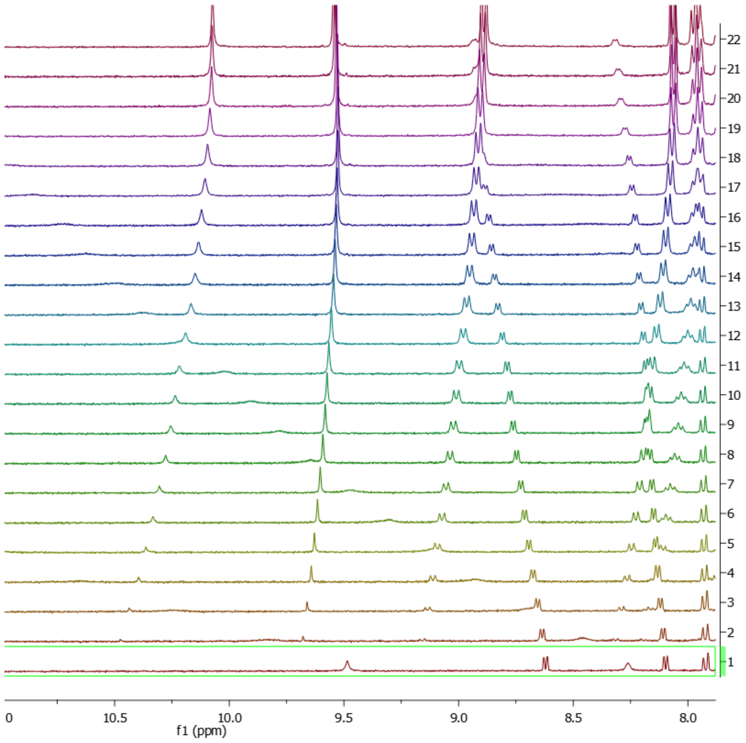

Figure S12. Stacked ^{1}H NMR (400 MHz, 298 K), expanded into the aromatic region from titration experiment for co-system **1H⁺•4** in $CDCl_3$/ DMSO-d_6 (99:1).

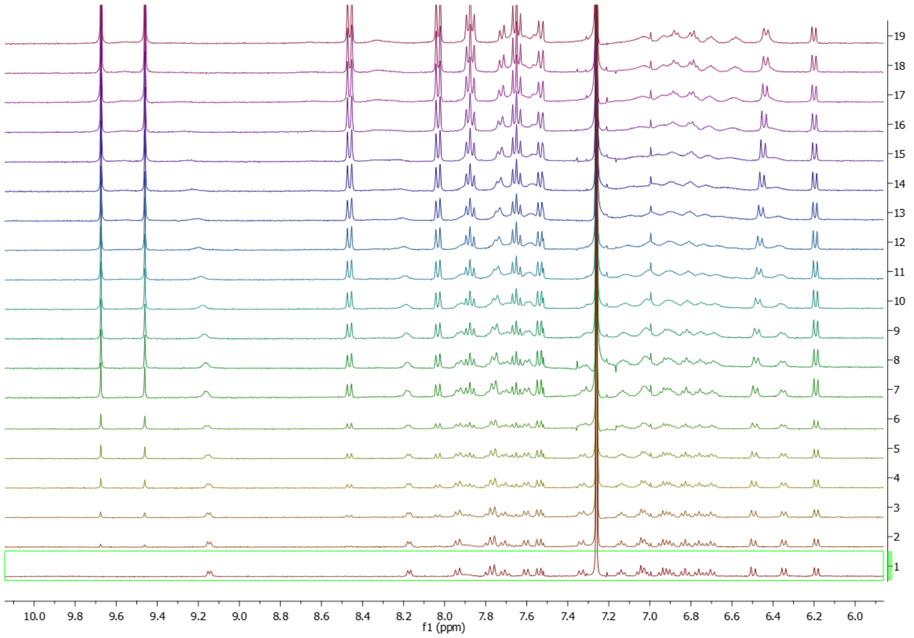

Figure S13. Stacked ^{1}H NMR (400 MHz, 298 K), expanded, from titration experiment for co-system **2H⁺•3** in $CDCl_3$/ DMSO-d_6 (99:1).

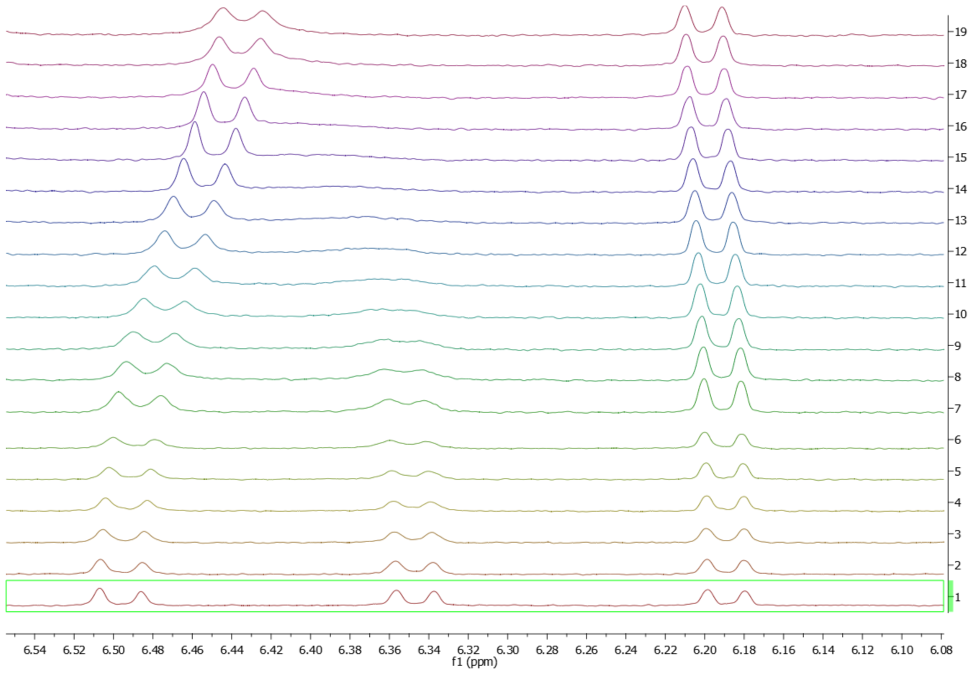

Figure S14. Stacked ^{1}H NMR (400 MHz, 298 K), expanded into 6.0-6.5 ppm, from titration experiment for co-system **2H+•3** in $CDCl_3$/ DMSO-d_6 (99:1).

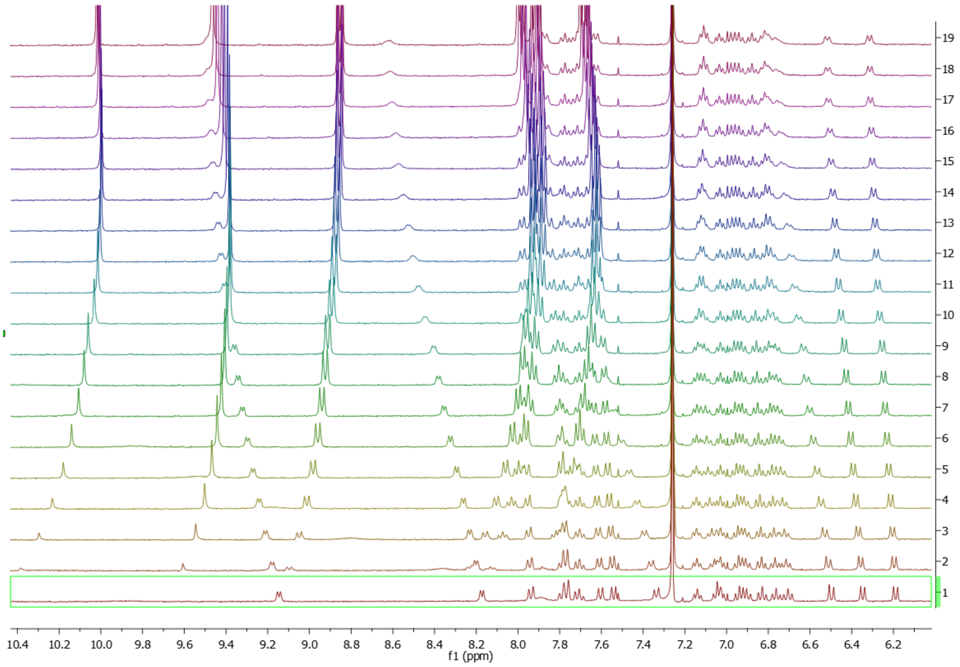

Figure S15. Stacked ^{1}H NMR (400 MHz, 298 K), zoomed in, from titration experiment for co-system **2H^{+}•4** in $CDCl_3$/ DMSO-d_6 (99:1).

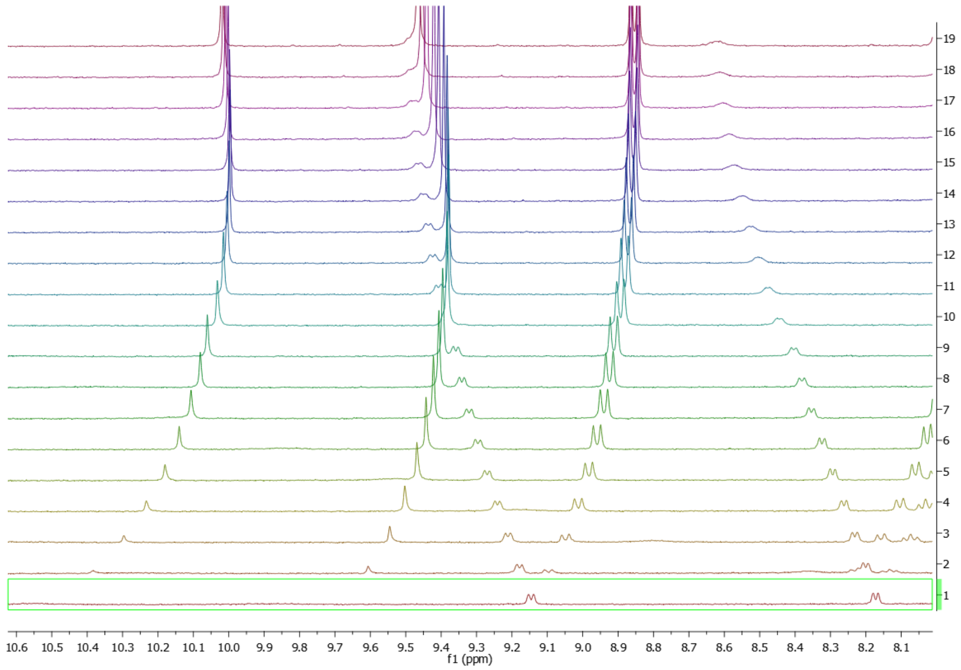

Figure S16. Stacked ^{1}H NMR (400 MHz, 298 K), expanded from 8.0-10.5 ppm, from titration experiment for co-system **2H^{+}•4** in $CDCl_3$/ DMSO-d_6 (99:1).

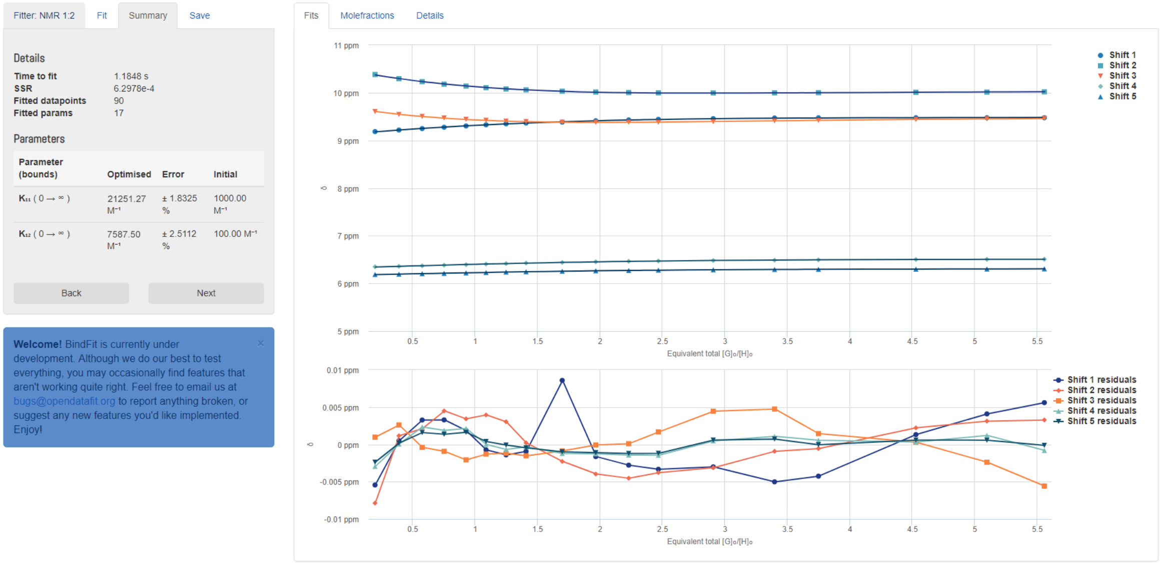

Figure S17. Results from the ^{1}H NMR titration studies of co-system **2H⁺•4** in $CDCl_3$/ DMSO-d_6 (99:1).

K_{11}=2.1 x 10^4 M^{-1} ± 1.8% K_{12}=7.6 x 10^3 M^{-1} ± 2.5%

Link to BindFit: http://app.supramolecular.org/bindfit/view/c9af70a6-f8db-4e99-a912-32998e91387b

S4: UV-vis Absorption Data

UV-Vis absorption spectroscopy titrations were performed in solutions of $CHCl_3$/ DMSO (99:1) with small aliquots of guest (**3** or **4**) added to host (**1**, **1H**$^+$, **2**, or **2H**$^+$). Bindfit modelling was performed on all systems. Each system was evaluated for the best 1:1 and 1:1/1:2 binding models based on percent error, the quality of the fitted data, and the randomness of the residual data (versus systematic error). Complexes **1•3** and **1H**$^+$**•3** illustrated higher error than acceptable and as such this data was alternatively treated SIVVU modelling which gave a better fit. The output were comparable to the Bindfit results but R^2 was much more accurate. We furthermore included 1:2 binding models because these types of systems have been reported in the past.[S4] We have also included an example of a potential 1:2 binding geometry (Below, fig S18).

Binding models were refined using http://app.supramolecular.org/bindfit/ and http://sivvu.org

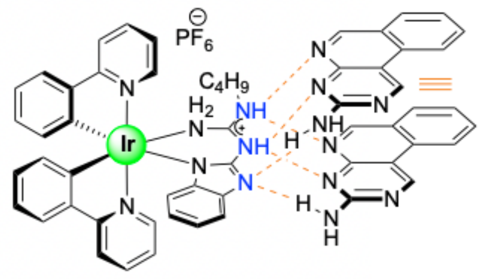

Figure S18. Proposed 1:2 intermolecular association geometry, in which **1H**$^+$ vertically bifurcates 2 molecules of **3** which are pi-stacking.

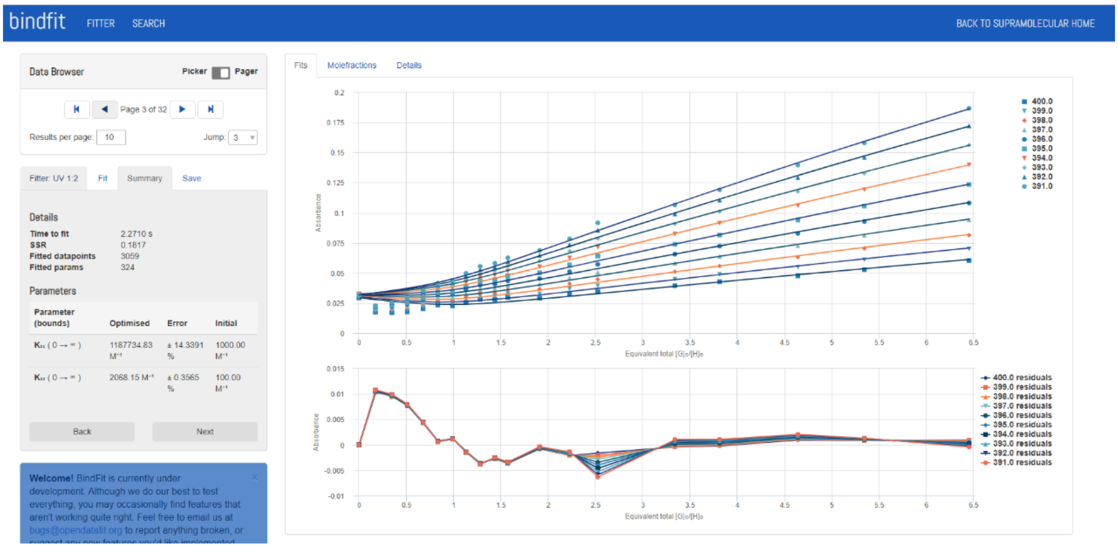

Figure S19. Results from the UV-vis absorption titration studies of co-system **1H^{+}•3** in $CHCl_3$/ DMSO (99:1).

K_{11}=1.1 x 10^6 M^{-1} ± 14% K_{12}=2.1 x 10^3 M^{-1} ± 0.4%

Link to BindFit: http://app.supramolecular.org/bindfit/view/7b10c11e-0d5f-4a6a-ba21-ffd1ca095ae9

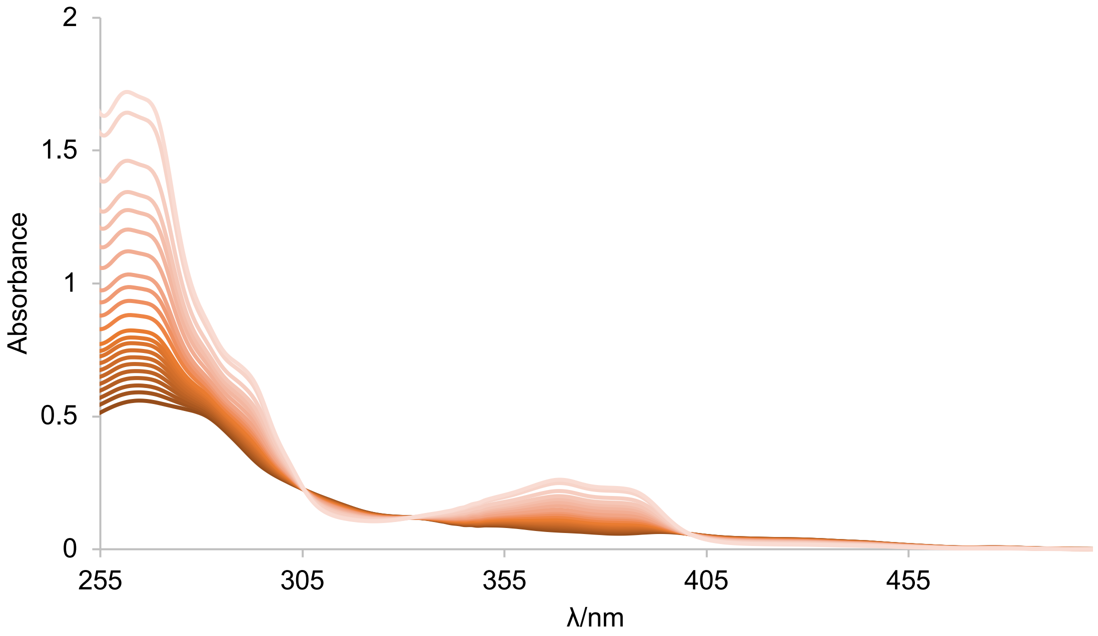

Figure S20. Results from the UV-vis absorption titration studies of co-system **1H^{+}•3** in $CHCl_3$/ DMSO (99:1).

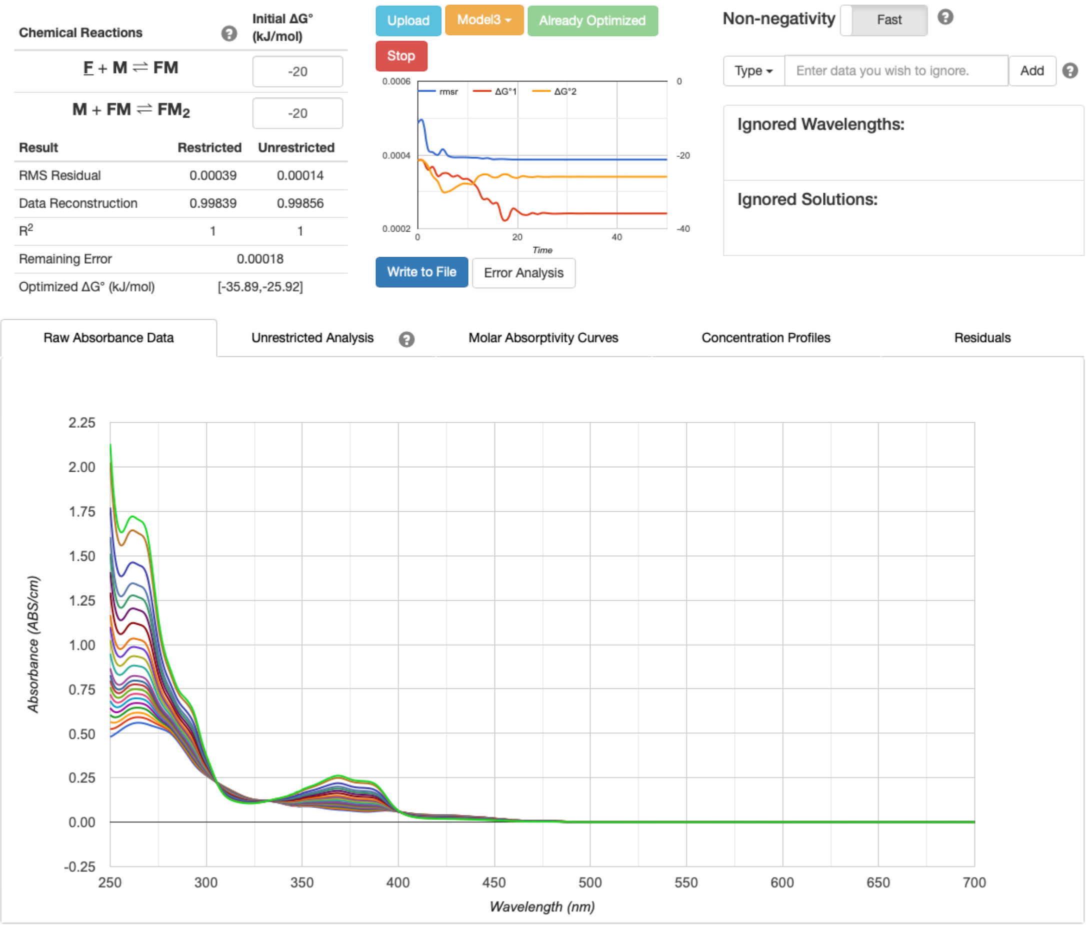

Figure S21. Results from the UV-vis absorption titration studies of co-system **$1H^+$•3** in $CHCl_3$/ DMSO (99:1) modelled using SIVVU.org with better fit.

Calculated constants from modelled energies:

K_{11}=1.9 x 10^6 M^{-1} K_{12}=3.4 x 10^4 M

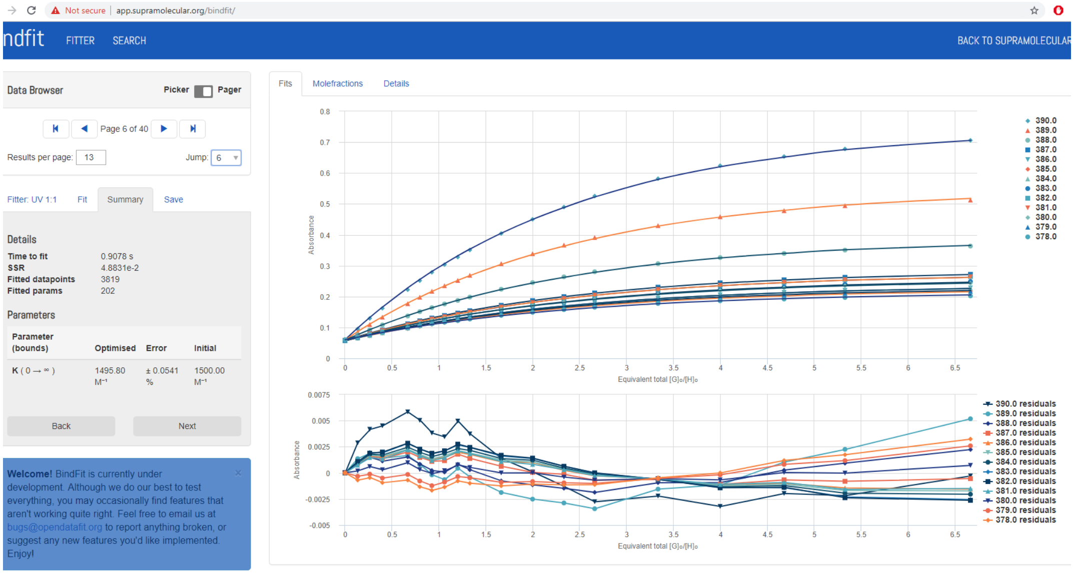

Figure S22. Results from the UV-vis absorption titration studies of co-system **1H$^+$•4** in $CHCl_3$/ DMSO (99:1).

$$K_a=1.5 \times 10^3\ M^{-1} \pm 0.05\%$$

Link to BindFit: http://app.supramolecular.org/bindfit/view/94dd3175-1285-4c8b-8599-a14ee7114fb2

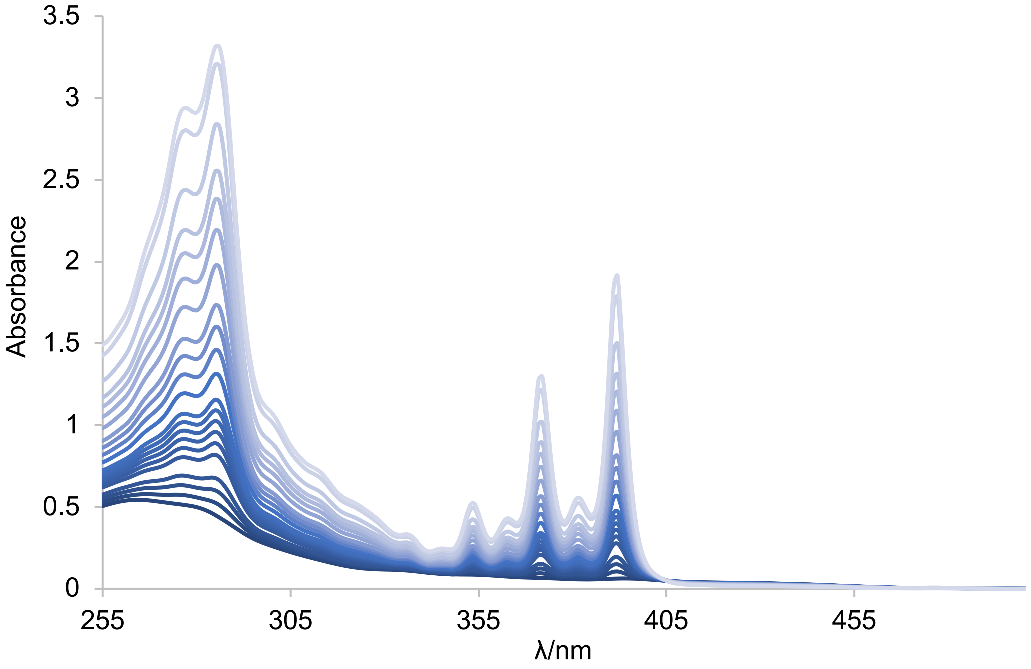

Figure S23. Results from the UV-vis absorption titration studies of co-system **1H$^+$•4** in $CHCl_3$/ DMSO (99:1).

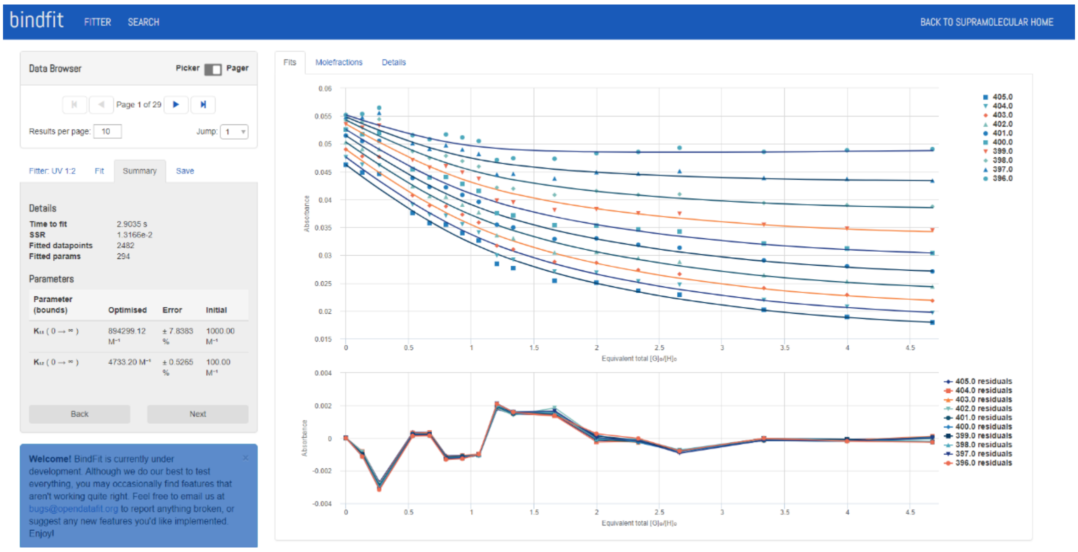

Figure S24. Results from the UV-vis absorption titration studies of co-system **1•3** in $CHCl_3$/ DMSO (99:1).

K_{11}=8.9 x 10^5 M^{-1} ± 7.8% K_{12}=4.7 x 10^3 M^{-1} ± 0.8%

Link to BindFit: http://app.supramolecular.org/bindfit/view/d25c5763-b8c6-444f-bcf2-efb13b67b230

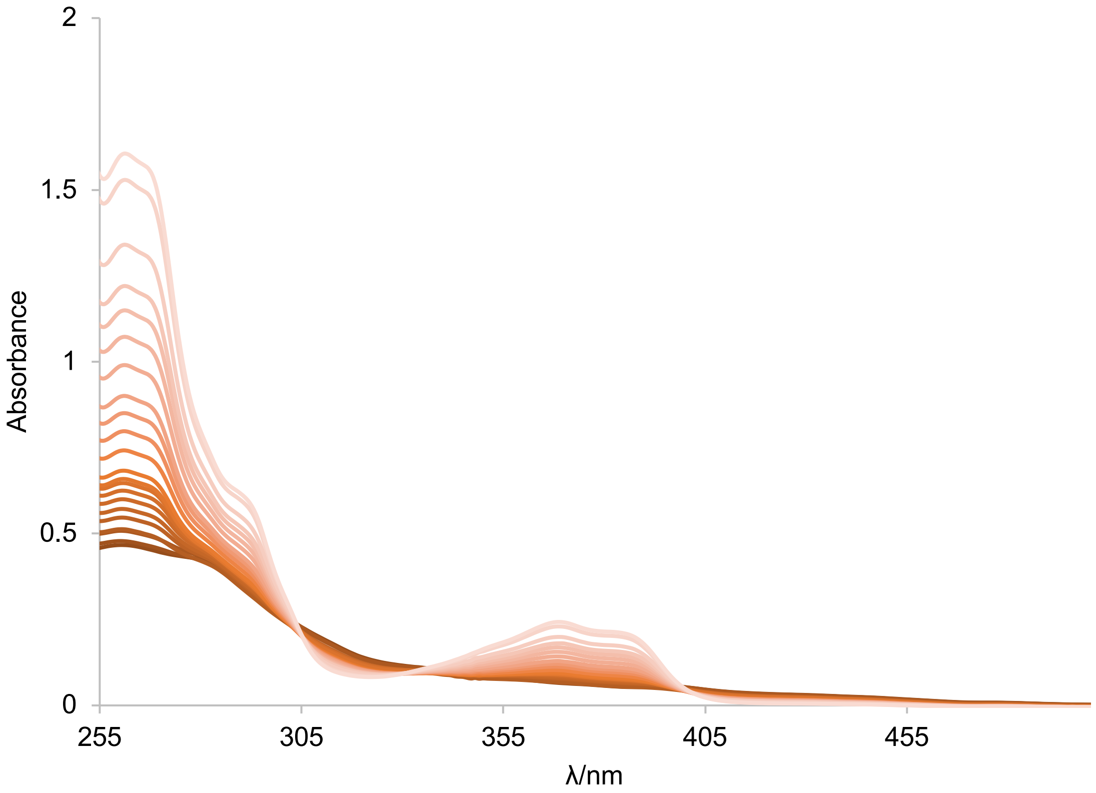

Figure S25. Results from the UV-vis absorption titration studies of co-system **1•3** in $CHCl_3$/ DMSO (99:1).

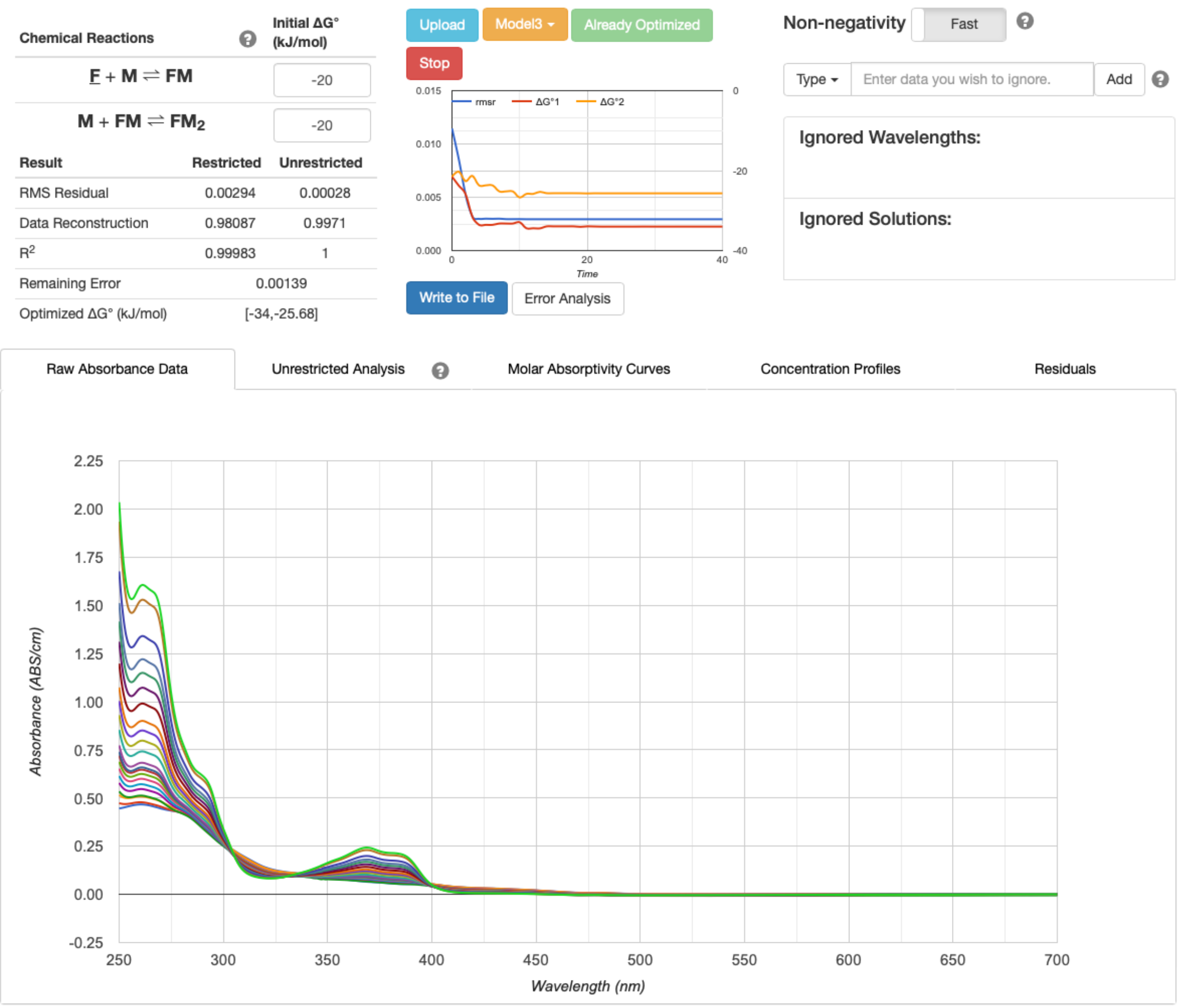

Figure S26. Results from the UV-vis absorption titration studies of co-system **1•3** in $CHCl_3$/ DMSO (99:1) modelled using SIVVU.org with better fit.

Calculated constants from modelled energies:

K_{11}=9.1 x 10^5 M^{-1} K_{12}=3.2 x 10^4 M

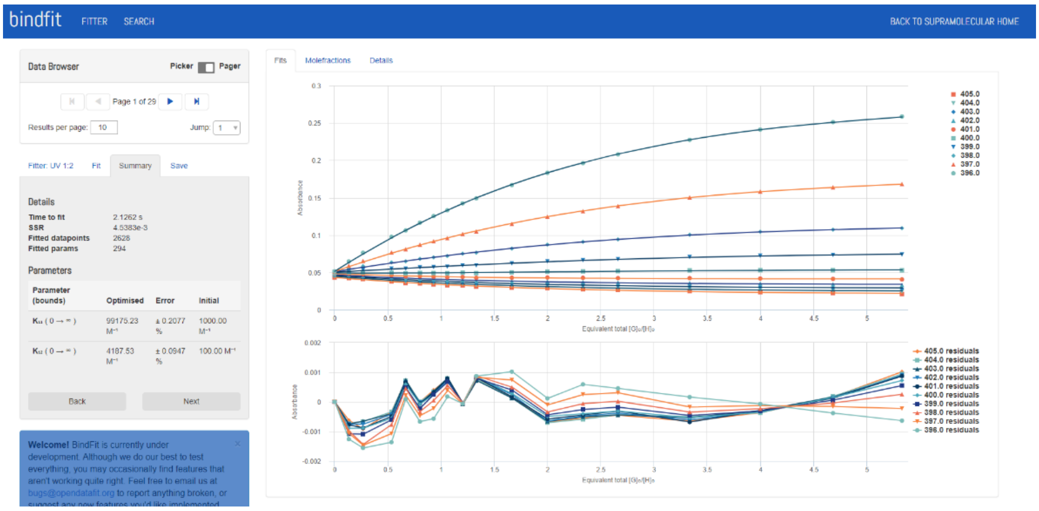

Figure S27. Results from the UV-vis absorption titration studies of co-system **1•4** in $CHCl_3$/ DMSO (99:1).

$K_{11}=9.9 \times 10^{4}\ M^{-1} \pm 0.2\%$ $K_{12}=4.2 \times 10^{3}\ M^{-1} \pm 0.1\%$

Link to BindFit: http://app.supramolecular.org/bindfit/view/64e93147-18ce-4cc3-8dfc-45fb7a766f52

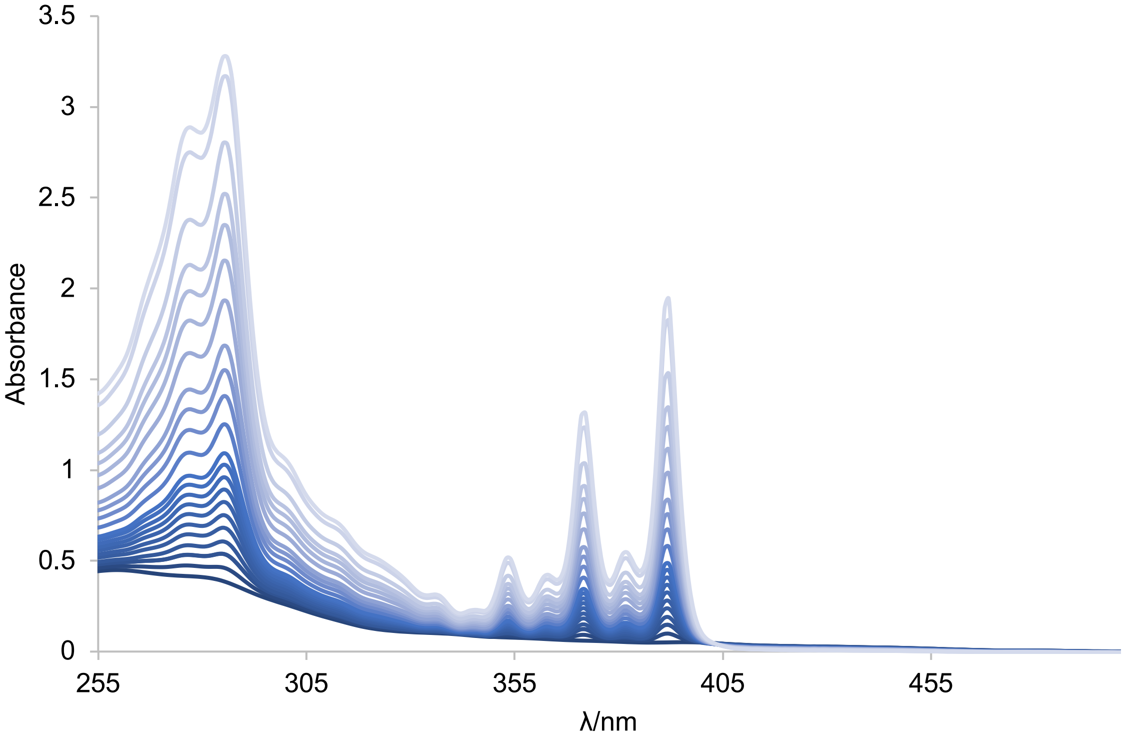

Figure S28. Results from the UV-vis absorption titration studies of co-system **1•4** in $CHCl_3$/ DMSO (99:1).

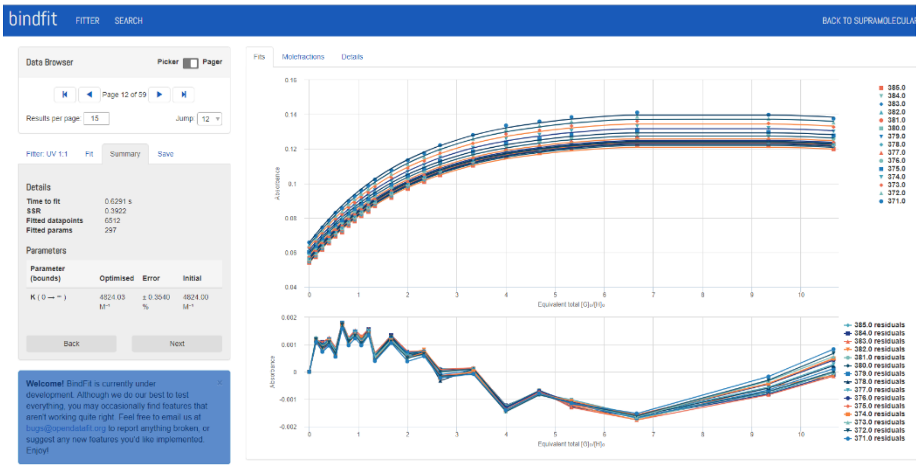

Figure S29. Results from the UV-vis absorption titration studies of co-system **2H$^+$•3** in $CHCl_3$/ DMSO (99:1).

$$K_a = 4.8 \times 10^3\ M^{-1} \pm 0.4\%$$

Link to BindFit: http://app.supramolecular.org/bindfit/view/e9e364b6-2db8-4e15-b74e-b48d636b60e0

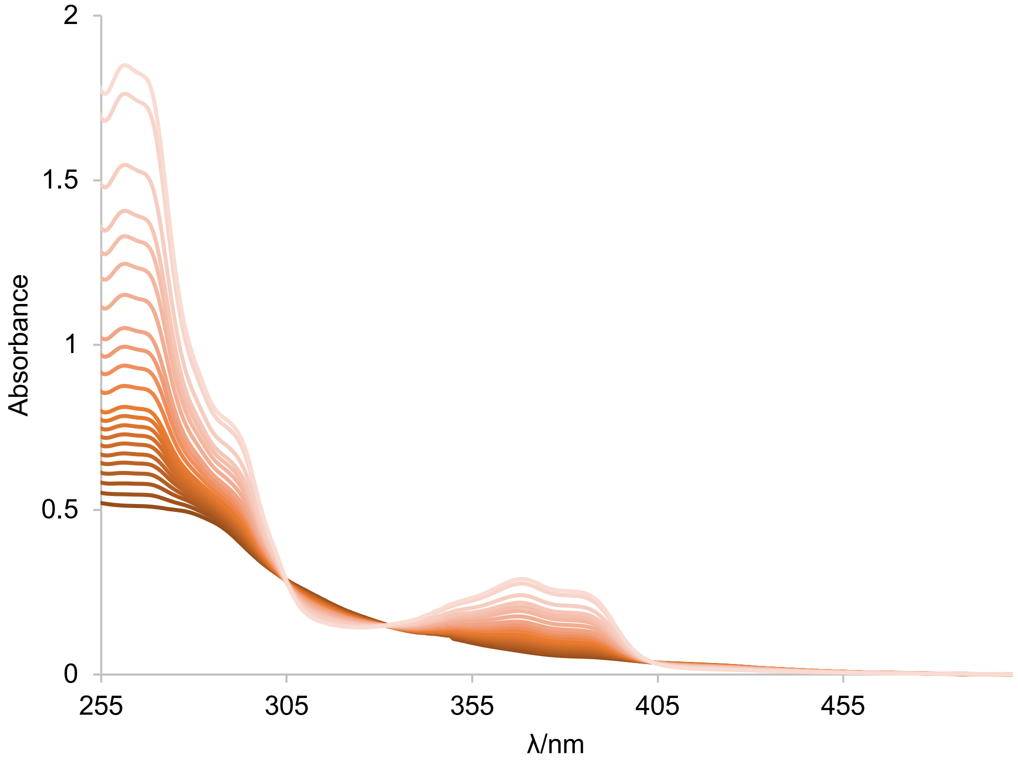

Figure S30. Results from the UV-vis absorption titration studies of co-system **2H$^+$•3** in $CHCl_3$/ DMSO (99:1).

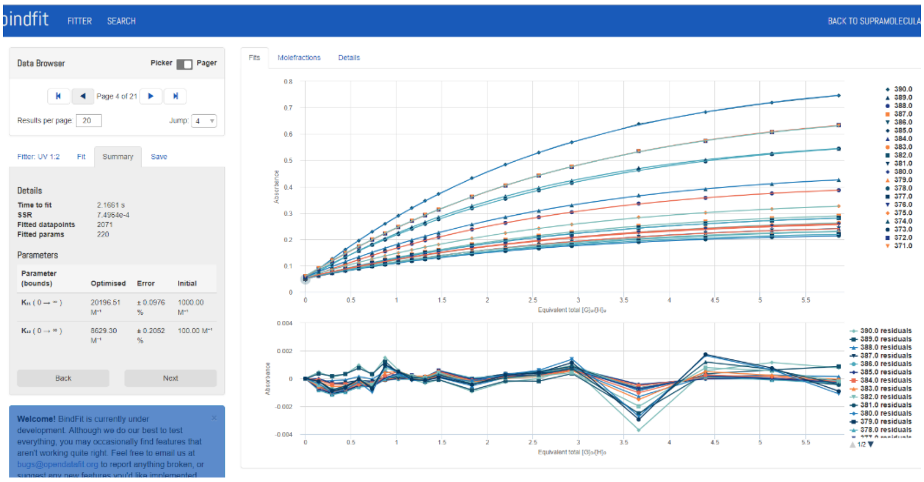

Figure S31. Results from the UV-vis absorption titration studies of co-system **2H^{+}•4** in $CHCl_3$/ DMSO (99:1).

$$K_{11} = 2.0 \times 10^{4}\ M^{-1} \pm 0.1\% \quad K_{12} = 8.6 \times 10^{3}\ M^{-1} \pm 0.2\%$$

Link to BindFit: http://app.supramolecular.org/bindfit/view/0ac4a9ac-4af6-4ee3-9e34-70499995765d

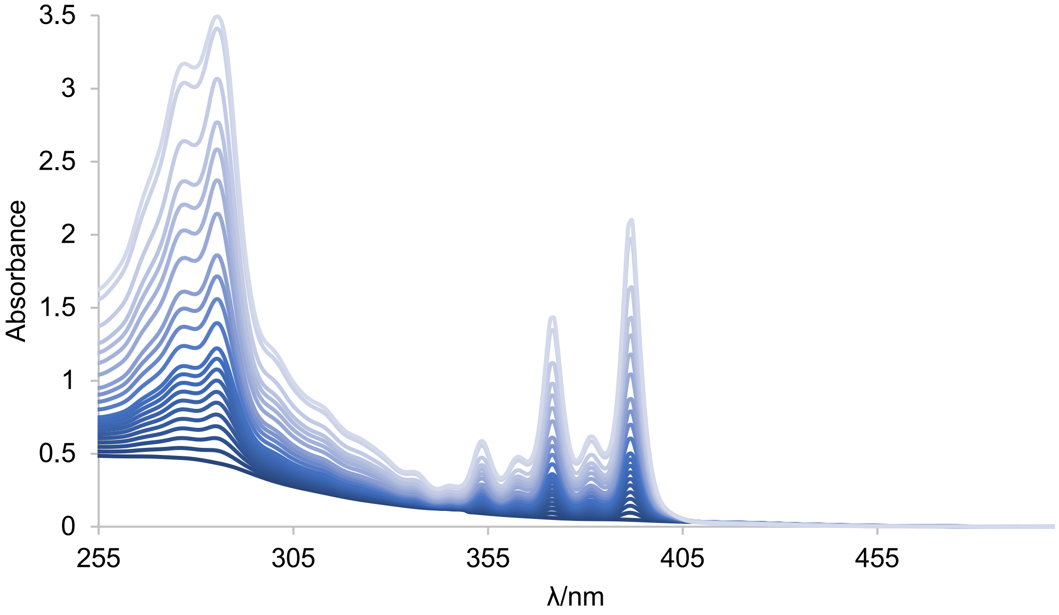

Figure S32. Results from the UV-vis absorption titration studies of co-system **2H^{+}•4** in $CHCl_3$/ DMSO (99:1).

References

[S1] Nonoyama, M. (1974) Benzo[h]Quinolin-10-Yl-N Iridium (III) Complexes. *Bull. Chem. Soc. Jpn.* 47, 767–768.
doi: 10.1246/bcsj.47.767

[S2] Balónová, B.; Martir, D. R.; Clark, E. R.; Shepherd, H. J.; Zysman-Colman, E.; Blight, B. A. (2018) Influencing the Optoelectronic Properties of a Heteroleptic Iridium Complex by Second-Sphere H-Bonding Interactions. *Inorg. Chem.* 57, 8581–8587.
doi: 10.1021/acs.inorgchem.8b01326

[S3] Balónová, B.; Shepherd, H. J.; Serpell, C. J.; Blight, B. A. (2020) IrIII as a Strategy for Preorganisation in H-Bonded Motifs. *Supramol. Chem.* 32, 1–12.
doi: 10.1080/10610278.2019.1649674

[S4] Blight, B.A.; Hunter, C.A.; Leigh, D.A.; McNab, H.; Thomson, P.I.T. (2011) An AAAA-DDDD quadruple Hydrogen Bond Array. *Nature Chem.* 3, 244-248.
